# Effects of lower extremity constraint-induced movement therapy on gait and balance of chronic hemiparetic patients after stroke: description of a study protocol for a randomized controlled clinical trial

**DOI:** 10.1186/s13063-021-05424-0

**Published:** 2021-07-19

**Authors:** Elaine Menezes-Oliveira, Gabriela da Silva Matuti, Clarissa Barros de Oliveira, Simone Ferreira de Freitas, Catia Miyuki Kawamura, José Augusto Fernandes Lopes, Ricardo Mario Arida

**Affiliations:** 1 Neurology/Neuroscience program, Federal University of São Paulo – UNIFESP, Botucatu street, 862 - 5° floor Edifico Ciências Biomédicas, São Paulo, Brazil; 2 Adults Physiotherapy Department, Associação de Assistência à Criança Deficiente, Professor Ascendino Reis avenue, 724 – Ibirapuera, São Paulo, Brazil; 3 Laboratory of Gait Analysis, Associação de Assistência à Criança com Deficiência – São Paulo, Professor Ascendino Reis avenue, 724 – Ibirapuera, São Paulo, Brazil

**Keywords:** Stroke, Constraint-induced movement therapy, Gait, Balance, Hemiparesis

## Abstract

**Background:**

Protocols involving intensive practice have shown positive outcomes. Constraint induced movement therapy (CIT) appears to be one of the best options for better outcomes in upper limb rehabilitation, but we still have little data about lower extremity constraint-induced movement therapy (LE-CIT) and its effects on gait and balance.

**Objective:**

To evaluate the effects of an LE-CIT protocol on gait functionality and balance in chronic hemiparetic patients following a stroke.

**Methods:**

The study adopts a randomized, controlled, single-blinded study design. Forty-two patients, who suffered a stroke, who were in the chronic phase of recovery (>6 months), with gait disability (no community gait), and who were able to walk at least 10 m with or without the advice or support of 1 person, will be randomly allocated to 2 groups: the LE-CIT group or the control group (intensive conventional therapy). People will be excluded if they have speech deficits that render them unable to understand and/or answer properly to evaluation scales and exercises selected for the protocol and/or if they have suffered any clinical event between the screening and the beginning of the protocol. Outcome will be assessed at baseline (T0), immediately after the intervention (T1), and after 6 months (T2). The outcome measures chosen for this trial are as follows: 6-min walk test (6minWT), 10-m walk test (10mWT), timed up and go (TUG), 3-D gait analysis (3DGA), Mini Balance Evaluation Systems Test (Mini-BESTest), and as a secondary measure, Lower Extremity Motor Activity Log will be evaluated (LE-MAL). The participants in both groups will receive 15 consecutive days of daily exercise. The participants in the LE-CIT group will be submitted to this protocol 2.5 h/day for 15 consecutive days. It will include (1) intensive supervised training, (2) use of shaping as strategy for motor training, and (3) application of a transfer package (plus 30 min). The control group will receive conventional physiotherapy for 2.5 h/day over 15 consecutive days (the same period as the CIT intervention). Repeated measures analyses will be made to compare differences and define clinically relevant changes between groups.

**Results:**

Data collection is currently on-going and results are expected in 2021.

**Discussion:**

LE-CIT seems to be a good protocol for inclusion into stroke survivors’ rehabilitation as it has all the components needed for positive results, as well as intensity and transference of gains to daily life activities.

**Trial registration:**

www.ensaiosclinicos.gov.brRBR-467cv6. Registered on 10 October 2017. “Effects of Lower Extremities - Constraint Induced Therapy on gait and balance function in chronic hemipretic post-stroke patients”.

**Supplementary Information:**

The online version contains supplementary material available at 10.1186/s13063-021-05424-0.

## Background

Cardiovascular disease is the leading cause of death in the world, representing 31% of the total number of deaths in 2017 [1]. Stroke accounts for almost half of these deaths [1] which means that it is the second greatest cause of death around the world [2] and the third most common cause of disability [3]. Hemiplegia is often the most common sequel caused by stroke, compromising independence in mobility at home or in the community, which sometimes results in losing premorbid society roles and requiring care for a long period of time [4].

Studies have reported that 6 months after the injury, 30% of patients are still unable to walk without assistance [5–7], and 1 year after the event (with relatively good recuperation), half of these patients are still not able to complete the 6-min walk test, walking just 40% of the predicted distance [7]. Despite all rehabilitative efforts, 35% of patients with initial paralysis in lower limbs are still unable to recover a functional gait and 25% are not able to walk without external aid [8]. Thus, within physiotherapy services, the majority of interventions involve approaches to gait training [9].

Protocols involving intensive practice have shown positive outcomes. Constraint induced movement therapy (CIT) appears to be one of the best options for better outcomes in upper limb rehabilitation. Experimental studies in the 1960s using CIT demonstrated that monkeys that suffered sensory deafferentation of their forelimb and then acquired learned non-use were able to use that paw again after having their unimpaired limb constrained for a number of days [10].

A growing number of studies have since supported the efficacy of CIT in upper limb rehabilitation for patients with chronic hemiparesis caused by stroke, which has been recognized and recommended within the treatment sets for this population [11–13]. Moreover, it has been considered the most effective physiotherapy approach for getting better rehabilitation outcomes for paretic upper limbs [14, 15].

CIT has been defined as a “therapeutic package” consisting of different numbers of compounds of combined treatment, used in a systematic and integrated way to engage the patient in using their affected limb for many hours per day over 2–3 consecutive weeks [16]. One of the main advantages of CIT in relation to the various different approaches used in neurological rehabilitation is that it is focused on the behavioral aspects of the method (monitoring, self-efficacy, solving problems, and contractual intervention); this guarantees the active participation of patient during the entire protocol [16].

The current CIT protocol consists of 3 main elements with multiple components and sub-components: (1) repetitive and task-oriented training (diary training with supervision), (2) behavioral strategies (transference package), and (3) constraint of affected limb (for upper extremity protocol) and/or any method to constantly remind the participant to use their more affected limb [16–18].

Post-stroke patients submitted to the CIT protocol for upper extremities present notable changes in the central nervous system (CNS) with improvement in cortical activation and increase of brain areas, using transcranial magnetic stimulation [19–21] or functional magnetic resonance [22–24].

There are still few data about lower extremity constraint-induced movement therapy (LE-CIT). In 2013, a case series was published which had been conducted on multiple sclerosis patients with a 4-year follow-up. At the end of the protocol they observed that these patients showed a notable improvement in *Lower Extremity Motor Activity Log* (LE-MAL) [25].

Although the studies used modified CIT, its methodology was not fully applied. For instance, the intensity applied was lower than that defined by the protocol; the presence of physical constraint on the non-affected side is described (this was discarded as it can create a bigger asymmetry and more abnormal movement); structure of training built without citing shaping (approaching in small steps); adoption of only one exercise, or a simple combination of different therapeutic approaches such as Bobath, muscle strengthening, or climbing stairs [26]; absence of a transference package, or differences in its structure (making only one homework list with exercises instead of a new list every day with different functional tasks); absence of a behavioral contract; and control group not receiving the same intensity of training [27, 28].

Despite not adhering exactly to the recommended model of CIT, these studies observed positive results such as an improvement in motor function, mobility, dynamic balance, discharge weight symmetry, gait ability, gait speed, length and width of step, and force of foot ground contact [26, 28]. However, in view of the above information, the investigation of the effects of the original LE-CIT protocol on gait functionality and balance of chronic hemiparetic patients following a stroke was not completely clarified.

The following research question was established to examine the effects of LE-CIT *vs* intensive conventional therapy on gait functionality and balance, as well as the transference of these gains in therapy to the environment outside the clinical setting in chronic hemiparetic patients following a stroke: is LE-CIT more effective compared with intensive conventional therapy with regard to gait functionality and balance in people suffering from stroke?

## Methods

### Study design

The study adopts a randomized, controlled, single-blinded study design in people suffering from stroke in the chronic stage of recovery. The study has been approved by the local ethics committee at *Associação de Assistência à Criança Deficiente (AACD)* (CAAE: 78269417.9.0000.0085, n° 2.478.704). The protocol will be conducted in the Adults Physiotherapy Department in the Rehabilitation Center of AACD, located at Av. Professor Ascendino Reis, 724, Ibirapuera, São Paulo, SP. All participants and their relatives and/or caregivers will provide written informed consent if they are accepted into the study. The study was registered with www.ensaiosclinicos.gov.br (Register Number: RBR-467cv6).

**Fig. 1:**
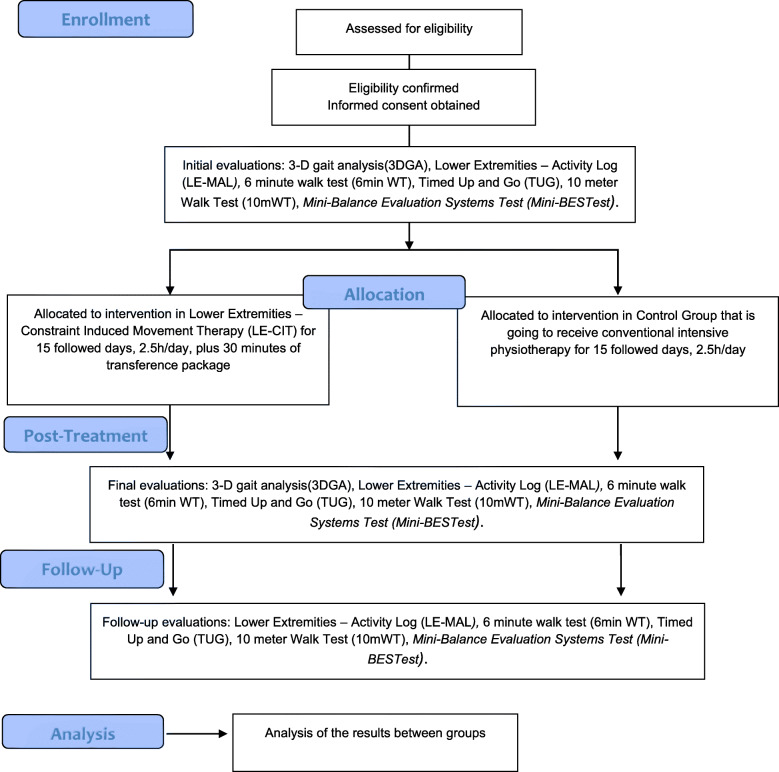
Consolidated Standards of Reporting Trials (CONSORT) flow diagram

A Consolidated Standards of Reporting Trials (CONSORT) flow diagram of the trial is shown in Fig. 1. and a Checklist of Recommended items to address in a clinical trial protocol and related documents (SPIRIT) is provided in a file.

### Population

The study population consists of people who had a stroke and want to improve their gait and balance ability. To minimize the chance that improvements occur as a result of spontaneous recovery, only participants who are in the chronic stage of recovery (>6 months after stroke) will be included in the study. The patients will be selected from initial multiprofessional evaluations at AACD. The physicians and physiotherapists will receive a checklist containing the inclusion criteria for this research. The patients who fulfill the criteria will be evaluated by an independent evaluator who is responsible for the screening.

Inclusion criteria for participants: (*i)* Medical diagnoses of stroke (ischemic or hemorrhagic); (*ii*) stroke at least 6 months before study participation; (*iii*) clinically stable; (*iv*) hemiparesis caused by stroke; (*v*) gait deficit caused by stroke (patients perception); (*vi*) able to begin hip and knee flexion on the affected side; (*vii*) able to move from sitting to standing independently even with aid or support of upper limbs; (*viii*) able to transfer while sitting independently; (*ix*) able to sustain body weight on the affected side even with the support of another person and/or aid; (*x*) able to walk at least 10 m with or without aid, with or without support of another person, barefoot; (*xi)* have only one or two main caregivers or relatives who live with the patient or spend substantial parts of the day with them; (*xii*) available to go to the rehabilitation center for 17 consecutive days and stay there for 3 h per day (15 days of treatment and the first day for initial tests and the last day for the final tests); (*xiii*) has not been subjected to orthopedic surgery for at least 6 months at the beginning of the protocol; (*xiv*) has not been subjected to chemical block for at least 3 months at the beginning of the protocol; and (*xv*) does not present an independent community gait (ability of walking alone in the community with or without gait advice at speed of ≤0.8m/s while walking).

Patients will be excluded who (*i*) do not accept the protocol for which they were randomly allocated; (*ii*) have speech deficits that render them unable to understand and/or answer properly to evaluation scales and exercises selected for the protocol; and (*iii*) have suffered any clinical event between the screening and the beginning of the protocol.

Patients will be removed from this trial who (*i*) have two consecutive absences without medical justification and (*ii*) have any clinical intercurrence that makes continuing with the exercises impossible.

Both patient and caregiver will be informed that their participation in this study protocol is totally voluntary and that they are free to discontinue participation at any time. They will also be informed that their treatment and care at AACD will not be affected, regardless of their decision regarding participation in the protocol, but during the 17 days of CIT they cannot enter into another concomitant therapy. The patient will be informed that the recorded data for the final analysis refer to evaluations and treatment applied to this study and that their identity will be always kept confidential. All volunteers will be informed about the potential types of discomfort, the risks, and the procedures employed in the investigation.

### Sample size and justification

After a sample size calculation, realized through a pilot study with 10 randomized patients in each group, using G*Power3.1.9.2 Software, we employed a matched-pair t-test *a priori*, using mean and standard deviation of Mini-BESTest (with an effect size of 0.93—sample size of 12 for each group) and 6minWT (with an effect size of 0.65—sample size of 21 for each group) as the primary outcome. Considering for analysis an α 0.05 and β 0.80, 21 patients were estimated for each group, totalizing 42 patients for this study.

### Randomization, blinding, and treatment allocation

#### Randomization procedure

Randomization will be realized through the allocation of vacancies (21 for each group) by a researcher who has no contact with the evaluation or intervention process. We created a spreadsheet with this sequence of allocation (intervention or control) and the distribution of the participants into the groups was made by sorting the order according to inclusion criteria and recruitment.

#### Blinding

To minimize evaluation bias, the evaluators responsible for screening and outcome evaluations will be blinded for the group that the patient belongs to. Moreover, the physiotherapist responsible for applying the protocols will not have contact with the test outcomes, but she will be aware of which group the patients belong to. Patients will not be informed to which group they will be allocated. They will probably be able to identify the treatment received, once it is written on the informed consent, but during the protocol they will be asked not to tell the evaluator which kind of treatment they have been submitted to.

#### Training of therapists and treatment of participants

The main evaluator is an experienced physiotherapist, trained for each chosen test and unaware of the patient’s group allocation. The physiotherapist responsible for applying the protocol (first author) has 10 years of experience with neurological patients; she has also received CIT training (for upper and lower extremities) in a total workload of 64 h and was trained by the creators of LE-CIT. She has been involved in clarifying and adjusting the protocol, making decisions and discussing specific cases.

#### Intervention group (LE-CIT)

The participants in this group will be submitted to physiotherapy 3 h/day for 15 consecutive days. The physiotherapy will be divided into 2 parts: the first 30 min will be dedicated to the transference package and 2.5 h of intensive training. The CIT protocol is composed of [16]:

1. Shaping—method based on principles of behavioral training in which the motor or behavioral aim is approached in small steps by successive approximation, which means that the task may be hampered by the patient’s motoric capacities or may stimulate the patient to perform the same activity faster. Every functional task is performed 10 times and feedback is given on each attempt. This activity is chosen from the shaping bank considering (a) specific articular movement where the main deficit lies, (b) the joint movement that is believed to have potential for improvement, and (c) preference for associating tasks that have a similar potential to produce specific improvements. Each shaping program is individualized and has 8–12 tasks selected from the shaping bank. However, new tasks may be created for each participant to improve their motor deficits. For instance, to improve weight-bearing on the paretic side you can ask the patient to move the non-affected limb to touch “X”s placed on the floor in front of their feet, to the side, and forward while standing. The therapist can record how many cycles the patient can complete over 30 or 45 s. When it gets easier (i.e., the quantity of completed cycles increases), the therapist can place the “X” a bit further away, or increase the balance demand (by reducing the base of support, or replacing the stable surface for an instable one such as a foam).
2. *Behavioral strategies of adherence (transference package):* These are strategies used to improve adherence through *Monitoring, Problem solving, and Behavioral contract.*

- ○ *Monitoring:* This involves strategies which lead the patient to observe and document their performance (activity mode, duration, frequency, effort perception, and physiological answer to the activity) through LE-MAL and home diary.
- ○ *LE/MAL and home diary:* In the daily administration of LE/MAL the patient answers questions about the participation of the impaired limb in daily activities, the period of reference being “from the last time that I asked you”.
- ○ *Problem solving:* The LE-MAL and home diary checking provides an opportunity to discuss which barriers were encountered when using the impaired limb in real life situations and to use this information to then find solutions (environmental and task adaptation). It has to be done every day during the protocol.
- ○ *Behavioral contract*: This is a formal agreement between the therapist, patient, and caregiver made on the first day. At this moment, the patient is committed to using their impaired limb during specific daily activities.
- ○ *Homework*: Patients choose 10 activities on the second day that they are going to try to perform at home: 5 that they believe will be easy to achieve and 5 that they believe will be more challenging. This list must be completed in about 30 min and will be revised during the first 30 min on the next day, every day during the protocol. For each activity, they must check if it was done or not, if they do not perform their task they must write (in the comments space) the reason for not completing the task. They are requested to conduct all the tasks listed; however, they are informed that they must complete at least 70% of the list. It is worth emphasizing that the homework list is part of the behavioral package which means transference of learned movements during therapy to daily life (within functional tasks).
- ○ *Schedule*: In this schedule the patient writes down the time spent on each activity in the list and how long they rested for.

#### Control group

The participants in this group will receive physiotherapy for 2.5 h/day over 15 consecutive days (the same period as the CIT intervention). The physiotherapist responsible for this group is experienced in stroke rehabilitation and has been working in this area for 10 years.

This conventional training can be defined as intervention without technological resources, using handling, verbal commands, positioning, gait training and/or pre-walking activities such as climbing stairs, balance training, lower limb strengthening, and other exercises that require standing and shifting weight to the impaired side [29].

### Measurements

Patient from both groups will be evaluated at 3 time points: T0 - 1° pre-treatment (1–3 days before the beginning of the protocol), T1 - 2° immediately after the end of the protocol (1–3 days after the last day), and T2 - 3° 6 months after the end of the protocol. All the evaluations will be conducted by an independent, blinded, experienced and trained evaluator (Table 1).

**Table 1:** Overview of measurements used in this study

Data	Time
**Demographics**	
Gender, age, time post stroke, type of stroke (ischemic or hemorrhagic), affected side, dominance	T0
**Walking performance measures**	
6min WT	T0, T1, T2
TUG	T0, T1, T2
10mWT	T0, T1, T2
**3-D**	T0, T1
**Balance measure**	
Mini-BESTest	T0, T1, T2
**Self-reported measure**	
LE-MAL	T0, T1, T2

As primary outcomes we have chosen Mini-BESTest and 6min WT, one test for gait performance and one test for balance evaluation.

For the follow-up evaluations, patients will receive, at the end of the protocol, a date to return. Between 30 and 15 days before the date scheduled, we will call them to remember about that. In case of giving up from the protocol we will use all the data collected until the moment of renunciation (it has been predicted at the consent form).

#### Demographics

At the first evaluation, characterization data will be recorded: gender, age, time post stroke, type of stroke (ischemic or hemorrhagic), affected side, and dominance.

#### Walking performance measures

To evaluate gait performance, we have chosen:

*6-min walk test (6min WT)* [30, 31]: This is used to characterize and monitor the gait changes in patients post stroke. It is commonly used to measure gait endurance and is a significant predictor for gait [32]. A systematic review published in 2017 showed that the clinically important difference (CID) for chronic hemiparesis patients varies from 28 to 42 meters [33].

*Timed up and go (TUG):* This is a simple test for functional mobility that requires the patient to move from sitting to standing, walking 3 m (as fast as possible), turning around (180°), walking back, and sitting again. The latency between letting go of backrest of the chair and then touching the backrest of the chair again after completing the walking is recorded. The best time of three attempts is to be used for the analysis. This is a common test used to evaluate the risk of falling and to monitor changes in patients’ mobility [34]. There is no defined value of CID for stroke patients; however, Hiengkaew et al. [35] shows that a change of ≥28% can indicate a relevant difference.

*10-m walk test (10mWT)*: This is a simple tool to quantify average speed during self-selected gait speed. The patient needs to walk in a hall with a 10-m demarcation, starting 1.5 m before the first mark and stopping 1.5 m after the last mark to exclude acceleration and slowdown. The stopwatch is started when the patient touches or crosses the first line and stopped when they touch or cross the last line. The CID for self-selected speed for stroke patients varies between 0.18 and 0.36 m/s (CI 95%) [35, 36].

*3-D gait analysis (3-DGA)*: This is performed at the Gait Laboratory. Subjects are equipped with skin-mounted reflective markers, placed on specific anatomical landmarks, as described by Kabada et al. [37]. Marker trajectories will be captured by an opto-electronic system consisting of eight infrared cameras (Qualisys OQUS300 system) operating at 100 Hz. Patients are instructed to walk barefoot at a self-selected speed along an 8-m walkway (26 feet). A minimum of six gait cycles for both lower limbs are collected, and a mean of these trials is obtained for the analysis and for consistency evaluation.

Kinematics are calculated according to a standard software procedure (Plugin Gait; Oxford Metrics, Oxford, UK) based on Kadaba et al. and Davis et al. [37, 38]. In order to improve the estimation of the thigh segment orientation, the Knee Alignment Device (KAD) was used during data collection.

From the kinematic data, the Gait Deviation Index (GDI) [39], the Gait Variable Scores (GVS), and Gait Profile Score (GPS) are calculated. The GDI is a multivariate measure of overall gait pathology. A GDI score equal to 100 or above indicates absence of gait pathology. Every 10-point decrease in GDI corresponds to one standard deviation from the mean of typically developing (TD) controls used during its calculation. The GVS is the root mean squared difference between a given kinematic variable calculated for a subject, and the mean of the same variable for a group of TD subjects. From all nine kinematic variables for which the GVS is calculated, an overall measure of gait pathology can be calculated (GPS). With all nine GVSs and the overall GPS, a Movement Analysis Profile can be plotted [40].

In a literature review, we found only one reference of minimal detectable change (MDC) for GDI scores related to post stroke patients. They suggest a MDC of 9.4 and 7.4 points for paretic and non-paretic limbs respectively [41]. For spatiotemporal parameters, we found these values for MDC: gait speed 14.61cm/s; stride length 11.96cm; cadence 8.58 step/min; step length 6.33cm; stance phase (StP) 3.60%; swing phase (SwP) 3.60%; and step width 2.47cm. For biomechanical parameters: hip maximum angle—SwP and StP 9.01° and 7.28°; hip minimum angle—SwP and StP 7.56° and 6.48°; knee maximum angle SwP and StP 6.54° and 4.93°; knee minimum angle SwP and StP 5.90° and 5.47°; ankle maximum angle SwP and StP 5.47° and 4.99°; ankle minimum angle SwP and StP 5.69° and 5.86° [42].

#### Balance measure

*Mini-Balance Evaluation Systems Test (Mini-BESTest)*: This is a tool used to evaluate balance control. It comprises 14 items divided into 4 subscales (anticipatory postural control, reactive postural control, sensory orientation, and gait stability). Each item is classified in an ordinal scale of 3 points (0 = severe and 2 = normal) and the maximum score is 28 points [43–45]. Tsang et al. [46] concluded that the MDC for this tool for post stroke patients is 3 points.

#### Self-reported measure

*Lower Extremity/Activity Log:* This is a structured interview, conducted by the therapist that evaluates how effectively subjects use their affected leg outside of the clinic setting in 14 common daily life activities on a scale that scores from 0 to 10 and quantifies functional performance (0 does not do and 10 does it normally) and the confidence with which the patient performs these activities (0 does not have confidence so does not do the task and 10 feels completely secure about doing the task). Patients are asked to score the quality of movement of the more-affected lower limb while performing the selected task, in the same way they are asked to score the confidence during this activity. LE/MAL has 3 dimensions: assistance scale, the functional ability scale, and the confidence scale. The assistance scale consists of 3 sub-scales: A, B, and C. Subscales A and B can take one of two forms, depending on the task. The form of subscale A (passive device-assistance) relevant to a given task can be either the orthotic subscale (A1) or the equipment modification subscale (A2). The scale for subscale B (self-initiated device-assistance) relevant to a given task can be either the assistive device subscale (B1) or the environmental support subscale (B2). Subscale C (person assistance) is the same for every item [25, 47, 48].

Instruments that are capable of measuring real-world/spontaneous use of lower limb are quite scarce. LE-MAL was not validated for Portuguese-Brazil language (our group is working on it). That is the reason for not using LE-MAL as one of our primary outcomes.

#### Data analyses

The software Excel (Microsoft) will be used to tabulate the data and the software SPSS will be used to do the statistical analysis. A significance level of 5% will be adopted.

We will use the Kolmogorov-Smirnov test to analyze the normality of the data. For the characterization of the sample and baseline comparison to test the homogeneity of the sample, the Qui-Quadrado will be used, but if we find statistical differences between the groups we will also analyze the clinical relevance of these differences.

The results will be compared in relation to the type of intervention and at the three timepoints proposed in this study (pre-treatment, post-treatment, and at 6 month follow-up) using the two-way analyses of variance, ANOVA, and the Bonferroni post-hoc test or Friedman, depending on the parameterization of the data.

To analyze the direction and magnitude of outcomes, we will use the Pearson or Spearman test of correlation.

The results will be described with mean and standard deviation for each group and mean and standard deviation of differences between groups and confidence interval.

## Results

For the execution of this trial, a period of 4 years has been predicted from the first patient recruitment to the last evaluation (6 month follow-up) of the last patient (Table 2). The participation of patients in this trial will last 7 months: initial evaluation, intervention, and 6 months after the end of protocol. The first study results are expected to be published by the end of 2021.

## Discussion

This paper describes the methodology of the first randomized controlled single-blinded clinical trial analyzing the effects of LE-CIT on the gait and balance of hemiparetic stroke survivors. Although studies on CIT for UE have been published over recent decades and a substantial body of evidence has been produced [11–15], LE-CIT has had little investigation. A few studies have been published, but these do not contain all the components proposed by the creator of the protocol. Recently, a description of LE-CIT was published which includes 4 pillars: (1) intensive supervised training delivered for 3.5h/day for 10 consecutive weekdays, (2) use of shaping as a strategy for motor training, (3) application of a transfer package, and (4) strongly encouraging the use of the more affected LE with improved coordination [47]. The same group has published the first study applying this version of the LE-CIT protocol to one patient with chronic hemiparesis, as a case report. In their study, the patient was submitted to LE-CIT for 10 consecutive days and by the end of the protocol an improvement was observed in LE-MAL(which infers the quality and confidence in using of the weaker LE) and balance score (measured by the Berg Balance Scale), as well as small changes in endurance and walking speed [49].

Our clinical trial proposal has high clinical significance for neurological rehabilitation, particularly for the stroke population. It is known that physical rehabilitation can be more effective than usual care or no attention in improving motor function, balance, and gait velocity [50]. Additionally, bigger doses of physical therapy provide significant benefits for motor function [50] and an average improvement of approximately 10% for both walking ability and activities of daily living [51].

Indeed, we believe that more important than motor advances is how far the patient can transfer these gains outside of the clinical setting, as improvements in clinical outcomes often do not translate to changes in community walking [52, 53]. It is believed that there is no “learned non-use phenomenon” related to lower extremities, instead “learned misuse” is attributed to these patients. Is that true? How can we explain that, even as their health improves, these patients continue not to walk as much as they are capable of doing? Ardestani (2019) suggests that perhaps the changes induced by the training in rehabilitation programs is just maintained in follow-up measures if there is greater paretic limb use to achieve increased daily stepping [54]. That is the reason why LE-CIT includes intensive supervised training linked with the transfer package; in this way, it can ensure that the motor gains will be incorporated into daily life activities [16, 47].

van Vliet et al. [55] also discuss the need for empowering stroke survivors in their own recovery since the health services will never have the capacity to ensure maximum motor function. This is a big challenge during the rehabilitation period and has been considered in the LE-CIT protocol by guaranteeing that the patient will be sufficiently engaged in using their more affected limb in daily activities. This will be achieved through the homework list (as part of the transference package). Alternatively, if the patient still finds a reason for not using the affected limb, they will then discuss this with the therapist in an attempt to find a solution to encourage the use of this limb in an efficient and confident way [16, 47].

Evidence from upper extremities supports the idea that patients who were subjected to CIT had better arm motor function [56] and greater use of the affected arm in daily life when compared with a control group, straight after the protocol and on follow-up assessments [57] (Table 2). This improvement provides additional evidence that CIT patients who receive the transfer package show significantly greater increases in gray matter in the hippocampus and sensory and motor areas when compared with a group that has not received it [57].

**Table 2:**
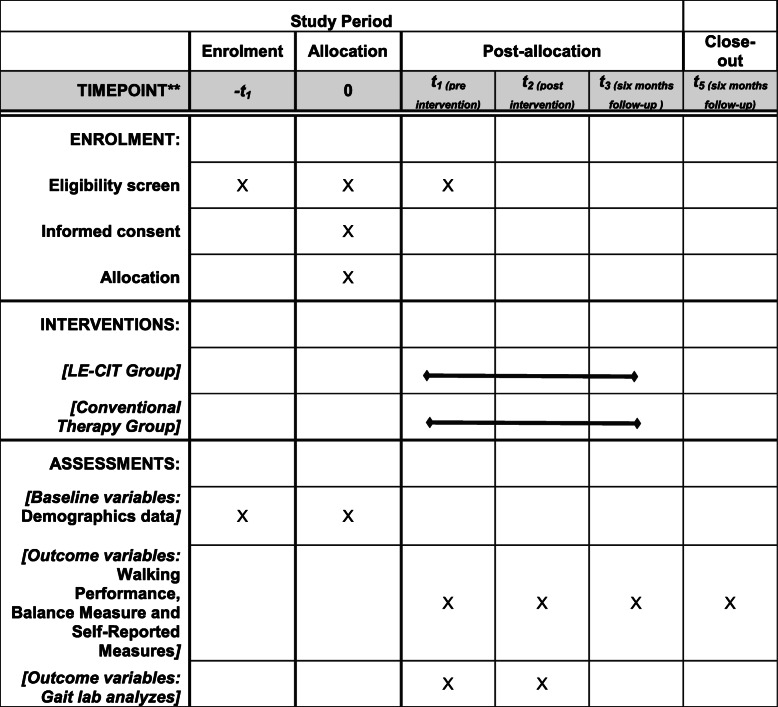
Schedule of enrolment, interventions, and assessments

The design of this clinical trial has strong and significant factors, such as the control group being exposed to the same number of hours and consecutive days of the protocol which means that the intensity and volume of training for both groups are similar. The similarity in the assessment of follow-up outcomes and the blinded evaluation for both groups is also a strong point of the trial’s design.

Another important aspect of our methodology is that we follow the initial proposal of the group that developed the protocol. It is important to clarify that when we first put this protocol into practice, we maintained the first study published by the group. At that time, the protocol was performed with 15 days of intervention [25]. Following information updates about the protocol from Dr Taub and colleagues at the University of Alabama in Birmingham to use 10 days of training, we decided to continue with the initial methodology plan as the protocol is still evolving and, in this way, offers us an opportunity to discuss the optimum amount of LE-CIT.

In conclusion this is the first clinical trial that proposes to evaluate whether the LE-CIT protocol can improve gait and balance outcomes, and whether these gains can be transferred to daily life activities. If our findings are positive, we will be able to suggest that this protocol may be better than the conventional intensive physiotherapy. However while intensity in rehabilitation is important, its transference to the real world and activities relevant to daily life may be even more valuable.

### Assessment of safety and adverse events

The main risks that the patient may be exposed to are related to the intensity of the protocol (fatigue and falls). Tiredness can be minimized by greater rest intervals between exercises if the patient shows fatigue. Regarding the risk of falls in exercises involving dynamic balance and gait training, we use a safety belt that is a device placed on the patient’s waist that facilitates the maintenance of standing posture in case of imbalance. For patients in the CIT group (who receive a homework list that can be carried out standing), responsible caregivers will be advised to wear this same belt at home (which will be provided as a loan on the first day of the protocol). Moreover, caregivers will receive very specific guidance on how to assist these patients at home during these tasks. Any adverse event must be reported on patients’ medical record, to ethical committee and in the clinical trial paper.

## Supplementary Information

**Additional file 1.**

**Additional file 2.**

**Additional file 3.**

**Additional file 4.**

**Additional file 5.**

**Additional file 6.**

**Additional file 7.**

**Additional file 8.**

**Additional file 9.**

## Data Availability

The datasets used and analyzed during the trial are available from the corresponding author on reasonable request.

## Abbreviations

LE-CIT: Lower extremity constraint-induced movement therapy
CIT: Constraint-induced movement therapy
T0: Baseline
T1: Immediately after the intervention
T2: 6 months’ follow-up
6minWT: 6-min walk test
10Mwt: 10-m walk test
TUG: Timed up and go
3DGA: 3-D gait analysis
Mini-BESTest: Mini Balance Evaluation Systems Test
LE-MAL: Lower Extremity Motor Activity Log
GDI: Gait Deviation Index
GVS: Gait Variable Scores
GPS: Gait Profile Score
TD: Typically developing
MDC: Minimal detectable change
StP: Stance phase
SwP: Swing phase
