# Supplementary material for: Effects of lower extremity constraint-induced movement therapy on gait and balance of chronic hemiparetic patients after stroke: description of a study protocol for a randomized controlled clinical trial
Source: Trials. 2021 Jul 19;22:463. doi: 10.1186/s13063-021-05424-0 (PMC8287769; doi:10.1186/s13063-021-05424-0)
Supplement: Supplementary file 1 — Additional file 1. [file 13063_2021_5424_MOESM1_ESM.docx]

**INFORMED CONSENT**

Main Researcher: Elaine Menezes de Oliveira

**Effects of Lower Extremities Constraint Induced Movement Therapy on gait and balance of chronic hemiparetic patients after stroke: a randomized controlled clinical trial.**

1. Introduction

You are being invited to be part of this clinical trial. This document is going to provide you all the needed information about how your therapy will be delivered and will help you to understand and decide whether you want to be part of this research protocol.

Please, read it carefully. Make all the questions about any unclear information and before making your decision discuss your main doubts with a relative, friend or health professional.

The participation in this research is totally voluntary. If you do not want, you do not have to. You will receive the best treatment being volunteer or not.

In case you decide to be part of this clinical trial we will ask you to provide us written informed consent. When you do that you are telling us that:

- You can understand what is written;
- You accept volunteering on this study;
- You accept the use of your personal and healthy information, as described.

This form has 5 pages. Make sure you have all of them. You will sign 2 copies, 1 will keep with the main researcher and the other one belongs to you.

**2. What is the aim of this study?**

The main objective is to evaluate if the participants will get better outcomes on gait and balance when submitted to a protocol that combine exercises for legs in an intensive way (2.5 hours/day) and engaging them to make tasks at home for transferring the gains from therapy to outside of clinical set.

3. What does participation in this research involve?

**Population:** Will be part of this study patients who suffered a stroke event, in the chronic phase of recovery (>6 months), with gait disability, with deficits in only one side of the body, clinically stable; available to go to the rehabilitation center for 17 consecutive days and stay there for 3 hours per day; with no more than 2 absences during the protocol, and that complies the treatment (the protocol will be defined through drawing).

Procedures:

The participants will be divided into 2 groups through drawing made by a person who is not part of the research:

Groups:

A) Lower Extremities - Constraint induced movement therapy.

B) Conventional Intensive Therapy.

**GRUP A (Lower Extremities - Constraint induced movement therapy)**

The participants in this group will be submitted to physiotherapy 3 hours/day for 15 followed days. The physiotherapy will be divided into 2 parts: the first 30 minutes will be dedicated to the transference package and 2.5 hours of intensive motor training.

Participants will perform a mean of 8 to 10 different tasks during the protocol that will be divided, 4 to 5 task every other day.

During the first 30 minutes of each day an interview will be conducted, asking about the daily activities and about the homework proposed on the previous day (tasks selected from a list). These activities are always tasks of daily lives (it will never be physical exercises), for instance, walking between the rooms of the house, dressing while standing, reaching a cabinet, etc. These attempts are daily checked. Suggestions are made, and whether necessary another attempt is given in a way to make the patient understand where are the mistakes and how to improve it for a well-done task.

**GRUP B (Conventional Intensive Therapy)**

The volunteers in this group will receive conventional physiotherapy for 2.5 hours/day over 15 followed days.

This conventional training can be defined as intervention without technological resources, using handling, verbal commands, positioning, gait training and/or pre-walking activities such as climbing stairs, balance training, lower limb strengthening and other exercises that require standing and shifting weight to the impaired side. It is quite similar to what you have already received or will receive here at AACD or any other rehabilitation center, but in an intensive way.

Both groups will receive physiotherapy with the same therapeutic aim, which means improving your gait and balance. Both groups will be exposed to the same risks (item 6). The main difference between the groups is about the structure of applying the exercises.

**MEASURES:** before you starting the protocol you will be submitted to an evaluation protocol.

These measures will be used to compare the effects of the therapies chosen on gait and balance. They will be performed 1-3 days before the beginning of the protocol, 1-3 days after its end, and 6 months after finishing the protocol.

1. **3-D GAIT ANALYSIS:** It will be performed on the laboratory of gait analysis at AACD. You will need to dress swimsuit (or short and top), some measures (as leg and foot length, height and weight). Subjects are equipped with skin-mounted reflective markers that will be captured by infrared cameras. You will be asked to walk about 10 times and it will spend about 45-60 minutes. That evaluation allows us to understand how well you are walking.
2. **WALKING PERFORMANCE MEASURES:** We will evaluate your gait speed in some tests performed at adults’ physiotherapy department (AACD): “6 minute walk test” (to measure you endurance and distance reached), “10 meter walk test” (to measure your gait speed while performing self-selected walking) and “*Timed up and go*” (to evaluate how fast you can walk and turn around). All these tests will spend about 40 minute.
3. **BALANCE EVALUATION:** We will evaluate your balance using the Mini-BESTest that measure your reactions in different balance situations as sitting to stand and walking.

THE MAIN DISCOMFORT YOU CAN EXPERIENCE ARE RELATED TO THE RISK OF FALLS (DESCRIBED ON ITEM 6). DURING THE TESTS WE WILL ALWAYS HAVE AN AUXILIAR EXPERIENCED ON AVOIDING FALLS DURING DINAMIC ACTIVITIES.

**Refund**

You are not going to be paid for being part of this study.

**4. What is going to happen with my outcome measures?**

The collected data will be analyzed by the therapists and the outcome will be compared between groups to find whether there are differences and on what way they happen on rehabilitation of people who suffered from stroke.

5. What are the main benefits?

You will not have any direct benefit by accepting being volunteer on this study, but you are going to receive physiotherapy that can make you improve your strength and movement of your legs, balance and gait.

6. What are the possible risks?

This is a study involving exercises that are going to be performed mostly while standing, the main risks are tiredness and falls.

Related to the risk of falls it will be reduced by the supervision of the therapist and the using of a transfer belt (during the therapy and at home) that on the verge of falling it can be avoided by the therapist or caregiver by holding the belt keeping the patient safe. In case it is not enough and the fall happens, the caregiver must note any sign of loss of consciousness, somnolence, vomiting (or nauseas), intense pain or any apparent injury; in case of presenting any of these sign the patient must be conducted to the nearly hospital for medical care and the main researcher must be contacted as soon as possible. The main researcher will be available for any doubts from medical team and/or the volunteer. In case this fall happens at AACD the emergency flow will be contacted and a doctor will immediately evaluate the patient and offer all the care needed (including hospitalization, whether necessary) with no costs for the patients.

Related to tiredness we can offer more time for resting between the exercises according to the patients’ complaint or loss of income.

For any other discomfort or clinical complication during the tests the procedure is the same, contacting the institutional emergency service. All the procedures of this study will happen under supervision and authorization of ethical committee from AACD and Federal University of São Paulo (UNIFESP), that follow the criteria of Ethics and Research with Human Beings as resolution nº. 196/96 of National Health Council.

**7. Do I have to be part of this study?**

Being part of any study protocol is a volunteer act. If you don’t want to be part of, you don’t have to. Whether you decide to volunteer and then you change your mind you are free to give up at any moment.

It is your decision whether should or not being part of that, or even give up. It will not affect your routine of treatment at AACD or your relationship with the ones who are going to treat you.

The proposed treatment at first evaluation at AACD will be maintained, and your participation (or even waiver) in this study will not affect that.

**8. What if I give up from this study?**

In case you decide to give up, the researchers would like to keep the data collected about your health status. It will help them make sure that the search results will be correctly measured. If you do not want to do this, you must inform them before entering the research project.

**9. Could this study be suddenly interrupted?**

The study will only be interrupted after the review and agreement of the Local Ethics Committee.

**10. How am I going to be informed about the outcomes of this study?**

The outcomes of this study will be provided on the final report of this research, and in case you want this information, the therapist can provide it to you. The results will be also published in a scientific journal. Because of the fact that the results must be analyzed maybe some time is necessary for these results becoming public, so we are going to provide an abstract written in Portuguese with objective and clear words for easy understanding.

After 6 months from the end of the protocol you will have the right of receiving the other protocol (for which you were not drawn). This decision will be made at your follow-up evaluation.

**11. What else do I need to know?**

- **How can I Access my data?**

You have the right of accessing and asking for clarification and revision of the collected data about you at any time during or after your participation in this study. We would like to remind you the confidentiality of all the collected data even when publishing the results of this study.

**12. Who can I contact?**

**For more information:**

If you need more information about this research project you can contact the main researcher Elaine Menezes de Oliveira (Phone number: +5511 5576-0924 from 7:00AM to 13:00PM on working days, other days and times available on cellphone number +5511 99853-3107 and/or by e-mail: [elaine_m_oliveira@yahoo.com.br](mailto:ELAINE_M_OLIVEIRA@YAHOO.COM.BR) or [emdoliveira@aacd.org.br](mailto:emdoliveira@aacd.org.br))

In case you cannot find her you can contact the physiotherapist on the same department (and second researcher of this study) Gabriela da Silva Matuti at the same phone number or +5511 98799-4439 and/or e-mail [gabrielamatuti@hotmail.com](mailto:gabrielamatuti@hotmail.com).

**For more details:**

If you have any question about the project and the way it is being conducted or any other doubt about being part of this research you can contact:

- CEP/AACD site to Av. Professor Ascendino Reis, 724. Ibirapuera – São Paulo – SP, Phone: +5511 5576-0472.

- CEP/UNIFESP site to Botucatu Street, nº 740, Vila Clementino, São Paulo/SP Postal Code: 04023-900. Personal or phone assistance: Monday, Tuesday, Thursday and Friday from 9 AM to 12 AM. E-mail: [cep@unifesp.br](mailto:cep@unifesp.br). Phone: +5511 5571-1062 or +5511 5539-7162.

Main Researcher: ELAINE MENEZES DE OLIVEIRA

I, .............................................……………………………………………………………………………………… ………………………………………. (name), ID: …………...................................... have read it and understood all the information about this study. I was informed about the procedures involved, including any expected or known inconveniences, risks or discomforts in potential and their implications as they are known to the researchers.

I do understand that my participation in this study allow the researchers to access to my medical history and I agree with that.

I give my permission to my medical team, hospitals or other laboratory outside of this institution to publish information about my disease and the treatment needed for this research project. I understand that this information will be maintained confidential.

I had the opportunity to make all the questions and I am satisfied with the answers. I understand that I will receive a copy of this document to keep with me, besides that the original copy signed by me will be archived by the researcher.

I freely choose to participate in this study and understand that I can withdraw at any time.

I agree to participate in this study.

|  |  | | |
| --- | --- | --- | --- |
| Participant signature | | | |
| Person name that conduct the process: | | | Date: |
| Signature: | | | |
| Witness's name: | | Date: | |
| Signature: | | | |
