# Supplementary material for: Effects of lower extremity constraint-induced movement therapy on gait and balance of chronic hemiparetic patients after stroke: description of a study protocol for a randomized controlled clinical trial
Source: Trials. 2021 Jul 19;22:463. doi: 10.1186/s13063-021-05424-0 (PMC8287769; doi:10.1186/s13063-021-05424-0)
Supplement: Supplementary file 3 — Additional file 3. [file 13063_2021_5424_MOESM3_ESM.pdf]

Activities to be "done with the help of someone else"

My therapist, ________________, and I have also agreed that I will emphasize use of my more-affected leg by doing the following activities with the help of someone else. I will receive help because: 1) safety would be a problem if I tried to do them independently, or 2) it is impossible for me to do them independently. Safety is always the first consideration. The approximate times at which I will carry out these activities are also listed. The amount of assistance should also be described using the following scale:

1 – Total Assistance; I expend < 25% effort
2 – Maximum Assistance; I expend 25-49 % effort
3 – Moderate Assistance; I expend 50-74% effort
4 – Minimal Assistance; I expend 75% or more effort
5 – Supervision/Setup; I require standby assist, cueing, item/device set-up

A.M. Activities done with the help of someone else and emphasizing use of my more-affected leg	Assistance	Asst. Device

P.M. Activities done with the help of someone else and emphasizing use of my more-affected leg	Assistance	Asst. Device

© 2014 UAB CI Therapy Research Group

Activities I will not do for safety reasons

After discussion with my therapist, ________________, I understand that I should avoid the following activities because doing them could be unsafe.

____

____

____

____

____

____

I,______________________, agree to abide by the above terms to the best of my ability in all situations when I am away from the treatment clinic, both at home and when I am away from home, including social situations.

______________________ ______________________

Signature of Patient Signature of Therapist

______________________ ______________________

Witness Signature of Caregiver

© 2014 UAB CI Therapy Research Group
