# Supplementary material for: Effects of lower extremity constraint-induced movement therapy on gait and balance of chronic hemiparetic patients after stroke: description of a study protocol for a randomized controlled clinical trial
Source: Trials. 2021 Jul 19;22:463. doi: 10.1186/s13063-021-05424-0 (PMC8287769; doi:10.1186/s13063-021-05424-0)
Supplement: Supplementary file 4 — Additional file 4. [file 13063_2021_5424_MOESM4_ESM.pdf]

Administration (check one and specify treatment day performed):
____ 1st (always done on treatment day one)
____ 2nd (treatment day ___)
____ 3rd (treatment day ___)

BEHAVIORAL CONTRACT
(for the Legs)

General

I,______________________________, agree to use my more-affected leg as much as possible during the treatment period, when in and outside of the treatment clinic, and to use the improved coordination that I am taught. The purpose of the emphasizing use of my more-affected leg is to: 1) re-wire my brain's ability to control my more-affected leg, 2) increase my skill with walking and balance activities, and 3) increase my confidence with using my more-affected leg and improving the coordination between my legs. I will emphasize use of my more-affected leg and improved coordination of both my legs at any time or for any task which I have agreed to do so. An exception will be that I will not try to use my more-affected leg alone or my improved coordination of both of my legs if my safety could in any way be affected; especially in the case of fatigue. Safety is always the first consideration.

Activities to be "done independently"

I agree to perform the following activities, when it is safe and possible to do so, independently in the home and outside the home, including social situations. I will attempt to use my more-affected leg and do so independently in all these activities, even if I had previously been using mostly my stronger leg or relying on others help for some of those tasks. The only activities for which I will not emphasize use of my more-affected leg and not do independently are those: 1) in which my safety would be affected, and/or 2) when a task can only be accomplished with the help of someone else. These specific activities will be discussed with the staff of the Project; but, in general, it

is important that I remember that safety and caution always must be considered first before trying to independently carry out a task emphasizing the more-affected leg or my improved coordination.

I have agreed with my therapist, _________________, that I will independently emphasize use of my more-affected leg while performing the following activities. The approximate times when I think these activities are most likely to be carried out are also listed. I will start emphasizing use of my more-affected leg when I wake up at about _______ a.m.

A.M. Activities done independently and emphasizing use of my more-affected leg	Times	Assistive Device

P.M. Activities done independently and emphasizing use of my more-affected leg	Times	Assistive Device

© 2014 UAB CI Therapy Research Group
