# Supplementary material for: Effects of lower extremity constraint-induced movement therapy on gait and balance of chronic hemiparetic patients after stroke: description of a study protocol for a randomized controlled clinical trial
Source: Trials. 2021 Jul 19;22:463. doi: 10.1186/s13063-021-05424-0 (PMC8287769; doi:10.1186/s13063-021-05424-0)
Supplement: Supplementary file 5 — Additional file 5. [file 13063_2021_5424_MOESM5_ESM.pdf]

Task:________________________ **Lower Extremity Shaping Data Sheet**

Subject________________________________ SID__________

Date:________________ TD:__________ Time:__________ Therapist:__________________

Shaping Task Description:

Starting position: supine ___ sidelying ___ sitting ___ standing ___ other (specify):

Equipment Used	Placement/Location
1.	
2.	
3.	

Instructions to Participant ____

____

Other Descriptors ____

Assistive Device____________________ Orthotic Device______________________

Assistance________________________ Coaching__________________________

Parameter Measured (ParaM):____

Parameter 1 varied(ParaV1)____

Parameter 2 varied (ParaV2)____

Parameter 3 varied (ParaV3)____

Trial	ParaM	QOM	ParaV1	ParaV2	ParaV3	Comments
Unit (e.g.cm)						
1						
2						
3						
4						
5						
6						
7						
8						
9						
10						

Home Skill Assignment Worksheet

Name:
Date:
Tx Day:

Activities to be tried (write in blanks provided):

	Attempted?	Time spent	Comments
1. ______________________	Y or N	______mins.	
2. ______________________	Y or N	______mins.	
3. ______________________	Y or N	______mins.	
4. ______________________	Y or N	______mins.	
5. ______________________	Y or N	______mins.	
6. ______________________	Y or N	______mins.	
7. ______________________	Y or N	______mins.	
8. ______________________	Y or N	______mins.	
9. ______________________	Y or N	______mins.	
10. ______________________	Y or N	______mins.	

© 2011 UAB CI Therapy Research Group
