# Supplementary material for: Effects of lower extremity constraint-induced movement therapy on gait and balance of chronic hemiparetic patients after stroke: description of a study protocol for a randomized controlled clinical trial
Source: Trials. 2021 Jul 19;22:463. doi: 10.1186/s13063-021-05424-0 (PMC8287769; doi:10.1186/s13063-021-05424-0)
Supplement: Supplementary file 6 — Additional file 6. [file 13063_2021_5424_MOESM6_ESM.docx]

| **Lower Extremity MAL Score Sheet** | | | | | | | | | |  |
| --- | --- | --- | --- | --- | --- | --- | --- | --- | --- | --- |
| **Subject's Name:** ________________________________ **Rater:** _______________________________ Day_______ Date:________ | | | | | | | | | |  |
|  |  |  |  |  |  |  |  |  |  |  |
|  | | | | | | | | | |  |
|  | | | | | | | | | |  |
|  | | | | | | | | | |  |
| **Item** | **Objective** | **Miscellaneous Detail** | **CI** | **A1/A2** | **B1/B2/B3** | **Assistance** | **Funcitional Performance** | **Confidence** | **If no, why?** |  |
|  |  |  |  |  |  |  |  |  |  |  |
| 1 | Walking Indoors | Short:_____ |  |  |  |  |  |  |  |  |
|  |  | Moderate: ______ | C1:______ | A1:______ | B1:______ |  |  |  |  |  |
|  |  | Long:______ |  |  |  |  |  |  |  |  |
| 2 | Walking Outdoors | Short:______ |  |  |  |  |  |  |  |  |
|  |  | Moderate: ______ | C1:______ | A1:______ | B1:______ |  |  |  |  |  |
|  |  | Long:______ |  |  |  |  |  |  |  |  |
| 3 | Climbing stairs (up & down) | Number of stairs in one direction: ______ | C1:______ | A1:______ | B3:______ |  |  |  |  |  |
| 4 | Stepping over (an) object(s) |  | C1:______ | A1:______ | B1:______ |  |  |  |  |  |
| 5 | Turning around when standing |  | C1:______ | A1:______ | B1:______ |  |  |  |  |  |
| 6 | Coming to stand from a chair | Type of chair: ______ | C1:______ | A2:______ | B2:______ |  |  |  |  |  |
| 7 | Coming to stand from a toilet |  | C1:______ | A2:______ | B2:______ |  |  |  |  |  |
| 8 | Getting in and out of bed |  | C1:______ | A2:______ | B2:______ |  |  |  |  |  |
| 9 | Getting in and out of bath/shower |  | C1:______ | A2:______ | B2:______ |  |  |  |  |  |
| 10 | Getting in and our of car |  | C1:______ | A2:______ | B2:______ |  |  |  |  |  |
| 11 | Opening a door & walking through doorway |  | C1:______ | A1:______ | B3:______ |  |  |  |  |  |
| 12 | Washing hands/grooming at sink while in standing |  | C1:______ | A1:______ | B3:______ |  |  |  |  |  |
| 13 | Reaching into cabinet/closet (above shoulder level); Done in standing |  | C1:______ | A1:______ | B3:______ |  |  |  |  |  |
| 14 | Retrieving an object(s) from the floor (from standing position) |  | C1:______ | A1:______ | B3:______ |  |  |  |  |  |
|  |  |  |  |  |  |  |  |  |  |  |
|  | C1: Personal Assistance Scale | B1: Assistive Device Scale | |  |  |  |  |  |  |  |
|  | A1: Orthotic Scale | B2: Upper Extremity Scale | |  |  |  |  |  |  |  |
|  | A2: Equipment Modification | B3: Environmental Support Scale | |  |  |  |  |  |  |  |
