# Supplementary material for: Effects of lower extremity constraint-induced movement therapy on gait and balance of chronic hemiparetic patients after stroke: description of a study protocol for a randomized controlled clinical trial
Source: Trials. 2021 Jul 19;22:463. doi: 10.1186/s13063-021-05424-0 (PMC8287769; doi:10.1186/s13063-021-05424-0)
Supplement: Supplementary file 7 — Additional file 7. [file 13063_2021_5424_MOESM7_ESM.docx]

LE MAL

1. Walking indoors

**____**Yes

Distance**:**

____Short distances only/ within room

____Moderate distances/ between adjacent rooms

____Long distances/ length of house, mall, etc.

**____**NO

____No opportunity

____Unable to perform

____Possibly able to perform, but does not try/avoids task

PERSONAL ASSISTANCE SCALE (C1)

0 Cannot do task or requires the help of 2 or more people

2 Can do task with help of 1 person who does MOST of the work

4 Can do task with help of 1 person who does HALF of the work

6 Can do task with help of 1 person who does LITTLE of the work

8 Can do task but requires SUPERVISION of 1 person

10 Can do task alone

ORTHOTIC SCALE (A1)

0 Cannot do task

1 HKAFO used

2 2 KAFOs used

3 1 KAFO and 1 AFO used

4 1 KAFO used

5 2 AFOs used

6 1 AFO used

7 2 FOs or 1 FO and shoe modifications used

8 1 FO used

9 Shoe modifications used

10 No device used

ASSISTIVE DEVICE SCALE (B1)

0 Cannot do task

1 Platform walker (rolling or standard) used

2 Rolling walker used

3 Standard walker used

4 2 forearm crutches used

5 2 quad canes used

6 2 straight canes used

7 1 hemi-walker used

8 1 forearm crutch or 1 quad cane used

9 1 straight cane used

10 No device used

2. Walking outdoors

**____**Yes

Distance**:**

____Short distances/ less than 20 feet

____Moderate distances/ 20 feet to 1 block

____Long distances/ 1 block or more

**____**NO

____No opportunity

____Unable to perform

____Possibly able to perform, but does not try/avoids task

PERSONAL ASSISTANCE SCALE (C1)

0 Cannot do task or requires the help of 2 or more people

2 Can do task with help of 1 person who does MOST of the work

4 Can do task with help of 1 person who does HALF of the work

6 Can do task with help of 1 person who does LITTLE of the work

8 Can do task but requires SUPERVISION of 1 person

10 Can do task alone

ORTHOTIC SCALE (A1)

0 Cannot do task

1 HKAFO used

2 2 KAFOs used

3 1 KAFO and 1 AFO used

4 1 KAFO used

5 2 AFOs used

6 1 AFO used

7 2 FOs or 1 FO and shoe modifications used

8 1 FO used

9 Shoe modifications used

10 No device used

ASSISTIVE DEVICE SCALE (B1)

0 Cannot do task

1 Platform walker (rolling or standard) used

2 Rolling walker used

3 Standard walker used

4 2 forearm crutches used

5 2 quad canes used

6 2 straight canes used

7 1 hemi-walker used

8 1 forearm crutch or 1 quad cane used

9 1 straight cane used

10 No device used

3. Climbing stairs (up and down) ___Number of stairs (one way)

**____**Yes

**____**NO

____No opportunity

____Unable to perform

____Possibly able to perform, but does not try/avoids task

PERSONAL ASSISTANCE SCALE (C1)

0 Cannot do task or requires the help of 2 or more people

2 Can do task with help of 1 person who does MOST of the work

4 Can do task with help of 1 person who does HALF of the work

6 Can do task with help of 1 person who does LITTLE of the work

8 Can do task but requires SUPERVISION of 1 person

10 Can do task alone

ORTHOTIC SCALE (A1)

0 Cannot do task

1 HKAFO used

2 2 KAFOs used

3 1 KAFO and 1 AFO used

4 1 KAFO used

5 2 AFOs used

6 1 AFO used

7 2 FOs or 1 FO and shoe modifications used

8 1 FO used

9 Shoe modifications used

10 No device used

ENVIRONMENTAL SUPPORT SCALE (B3)

0 Cannot do task

2 Leans heavily on 2 rails or 1 rail and assistive device

4 Leans heavily on 1 rail

6 Leans lightly on 1 rail or only uses assistive device on one side

8 Lightly touches rail for balance only

10 No rail or assistive device used

--------------------------------------------------------------------------------------------

**Functional Performance**

0 1 2 3 4 5 6 7 8 9 10

Cannot do Half Normal

Normal

----------------------------------------------------------------------------------------------

**Confidence**

0 1 2 3 4 5 6 7 8 9 10

None Moderate Complete

4. Stepping over object

**____**Yes

**____**No

____No opportunity

____Unable to perform

____Possibly able to perform, but does not try/avoids task

PERSONAL ASSISTANCE SCALE (C1)

0 Cannot do task or requires the help of 2 or more people

2 Can do task with help of 1 person who does MOST of the work

4 Can do task with help of 1 person who does HALF of the work

6 Can do task with help of 1 person who does LITTLE of the work

8 Can do task but requires SUPERVISION of 1 person

10 Can do task alone

ORTHOTIC SCALE (A1)

0 Cannot do task

1 HKAFO used

2 2 KAFOs used

3 1 KAFO and 1 AFO used

4 1 KAFO used

5 2 AFOs used

6 1 AFO used

7 2 FOs or 1 FO and shoe modifications used

8 1 FO used

9 Shoe modifications used

10 No device used

ASSISTIVE DEVICE SCALE (B1)

0 Cannot do task

1 Platform walker (rolling or standard) used

2 Rolling walker used

3 Standard walker used

4 2 forearm crutches used

5 2 quad canes used

6 2 straight canes used

7 1 hemi-walker used

8 1 forearm crutch or 1 quad cane used

9 1 straight cane used

10 No device used

--------------------------------------------------------------------------------------------

**Functional Performance**

0 1 2 3 4 5 6 7 8 9 10

Cannot do Half Normal

Normal

---------------------------------------------------------------------------------------------

**Confidence**

0 1 2 3 4 5 6 7 8 9 10

None Moderate Complete

5. Turning around when standing (whole body with movement of feet)

## ____Yes

**____**No

____No opportunity

____Unable to perform

____Possibly able to perform, but does not try/avoids task

PERSONAL ASSISTANCE SCALE (C1)

0 Cannot do task or requires the help of 2 or more people

2 Can do task with help of 1 person who does MOST of the work

4 Can do task with help of 1 person who does HALF of the work

6 Can do task with help of 1 person who does LITTLE of the work

8 Can do task but requires SUPERVISION of 1 person

10 Can do task alone

ORTHOTIC SCALE (A1)

0 Cannot do task

1 HKAFO used

2 2 KAFOs used

3 1 KAFO and 1 AFO used

4 1 KAFO used

5 2 AFOs used

6 1 AFO used

7 2 FOs or 1 FO and shoe modifications used

8 1 FO used

9 Shoe modifications used

10 No device used

ASSISTIVE DEVICE SCALE (B1)

0 Cannot do task

1 Platform walker (rolling or standard) used

2 Rolling walker used

3 Standard walker used

4 2 forearm crutches used

5 2 quad canes used

6 2 straight canes used

7 1 hemi-walker used

8 1 forearm crutch or 1 quad cane used

9 1 straight cane used

10 No device used

---------------------------------------------------------------------------------------------

**Functional Performance**

0 1 2 3 4 5 6 7 8 9 10

Cannot do Half Normal

Normal

---------------------------------------------------------------------------------------------

**Confidence**

0 1 2 3 4 5 6 7 8 9 10

None Moderate Complete

6. Come to stand from a chair

**____**Yes What type: _______________

## ____NO

____No opportunity

____Unable to perform

____Possibly able to perform, but does not try/avoids task

PERSONAL ASSISTANCE SCALE (C1)

0 Cannot do task or requires the help of 2 or more people

2 Can do task with help of 1 person who does MOST of the work

4 Can do task with help of 1 person who does HALF of the work

6 Can do task with help of 1 person who does LITTLE of the work

8 Can do task but requires SUPERVISION of 1 person

10 Can do task alone

EQUIPMENT MODIFICATION: CHAIR (A2)

0 Cannot do task

2 Used raised chair with armrests

4 Used raised chair without armrests

6 Used standard chair with armrests

8 Used standard chair without armrests

10 Used recliner or soft sofa

UPPER EXTREMITY SCALE (B2)

0 Cannot do task

2 Pushed heavily with both arms

4 Pushed heavily with only one arm or lightly with both arms

6 Pushed lightly with one arm only

8 Lightly touched for balance only

10 Stands without any upper extremity support

---------------------------------------------------------------------------------------------

**Functional Performance**

0 1 2 3 4 5 6 7 8 9 10

Cannot do Half Normal

Normal

---------------------------------------------------------------------------------------------

**Confidence**

0 1 2 3 4 5 6 7 8 9 10

None Moderate Complete

##

7. Come to stand from a toilet

**____**Yes

## ____NO

____No opportunity

____Unable to perform

____Possibly able to perform, but does not try/avoids task

PERSONAL ASSISTANCE SCALE (C1)

0 Cannot do task or requires the help of 2 or more people

2 Can do task with help of 1 person who does MOST of the work

4 Can do task with help of 1 person who does HALF of the work

6 Can do task with help of 1 person who does LITTLE of the work

8 Can do task but requires SUPERVISION of 1 person

10 Can do task alone

EQUIPMENT MODIFICATION SCALE: TOILET (A2)

0 Cannot do task

2 Used elevated toilet with 2 armrests or 2 grab bars

4 Used elevated toilet seat with 1 armrest or 1 grab bar

6 Used elevated toilet seat only or standard height toilet with 2 armrests or 2 grab bars

8 Used stadard height toilet seat with 1 armrest or grab bar

10 Used standard height toilet seat with no arm rests or grab bars

UPPER EXTREMITY SCALE (B2)

0 Cannot do task

2 Pushed heavily with both arms

4 Pushed heavily with only one arm or lightly with both arms

6 Pushed lightly with one arm only

8 Lightly touched for balance only

10 Stands without any upper extremity support

---------------------------------------------------------------------------------------------

**Functional Performance**

0 1 2 3 4 5 6 7 8 9 10

Cannot do Half Normal

Normal

--------------------------------------------------------------------------------------------- **Confidence**

0 1 2 3 4 5 6 7 8 9 10

None Moderate Complete

8. Getting in and out of bed

**____**Yes

____NO

____No opportunity

____Unable to perform

____Possibly able to perform, but does not try/avoids task

PERSONAL ASSISTANCE SCALE (C1)

0 Cannot do task or requires the help of 2 or more people

2 Can do task with help of 1 person who does MOST of the work

4 Can do task with help of 1 person who does HALF of the work

6 Can do task with help of 1 person who does LITTLE of the work

8 Can do task but requires SUPERVISION of 1 person

10 Can do task alone

EQUIPMENT MODIFICATION SCALE: BED (A2)

0 Cannot do task

5 Modified bed used

10 Standard bed used

UPPER EXTREMITY SCALE (B2)

0 Cannot do task

2 Pushed heavily with both arms

4 Pushed heavily with only one arm or lightly with both arms

6 Pushed lightly with one arm only

8 Lightly touched for balance only

10 Stands without any upper extremity support

---------------------------------------------------------------------------------------------

**Functional Performance**

0 1 2 3 4 5 6 7 8 9 10

Cannot do Half Normal

Normal

---------------------------------------------------------------------------------------------

**Confidence**

0 1 2 3 4 5 6 7 8 9 10

None Moderate Complete

##

9. Getting in and out of bath or shower

**____**Yes

## ____NO

____No opportunity

____Unable to perform

____Possibly able to perform, but does not try/avoids task

PERSONAL ASSISTANCE SCALE (C1)

0 Cannot do task or requires the help of 2 or more people

2 Can do task with help of 1 person who does MOST of the work

4 Can do task with help of 1 person who does HALF of the work

6 Can do task with help of 1 person who does LITTLE of the work

8 Can do task but requires SUPERVISION of 1 person

10 Can do task alone

EQUIPMENT MODIFICATION SCALE: TUB (A2)

0 Cannot do task

2 Used tub transfer bench

4 Used tub chair with rail

6 Used tub chair only

8 Used rail only

10 No equipment used

UPPER EXTREMITY SCALE (B2)

0 Cannot do task

2 Pushed heavily with both arms

4 Pushed heavily with only one arm or lightly with both arms

6 Pushed lightly with one arm only

8 Lightly touched for balance only

10 Stands without any upper extremity support

---------------------------------------------------------------------------------------------

**Functional Performance**

0 1 2 3 4 5 6 7 8 9 10

Cannot do Half Normal

Normal

---------------------------------------------------------------------------------------------

**Confidence**

0 1 2 3 4 5 6 7 8 9 10

None Moderate Complete

10. Getting in and out of car

**____**Yes

## ____NO

____No opportunity

____Unable to perform

____Possibly able to perform, but does not try/avoids task

PERSONAL ASSISTANCE SCALE (C1)

0 Cannot do task or requires the help of 2 or more people

2 Can do task with help of 1 person who does MOST of the work

4 Can do task with help of 1 person who does HALF of the work

6 Can do task with help of 1 person who does LITTLE of the work

8 Can do task but requires SUPERVISION of 1 person

10 Can do task alone

EQUIPMENT MODIFICATION SCALE: CAR (A2)

0 Cannot do task

2 Requires sliding board

5 Requires modified seat

10 No modifications required

UPPER EXTREMITY SCALE (B2)

0 Cannot do task

2 Pushed heavily with both arms

4 Pushed heavily with only one arm or lightly with both arms

6 Pushed lightly with one arm only

8 Lightly touched for balance only

10 Stands without any upper extremity support

---------------------------------------------------------------------------------------------

**Functional Performance**

0 1 2 3 4 5 6 7 8 9 10

Cannot do Half Normal

Normal

---------------------------------------------------------------------------------------------

**Confidence**

0 1 2 3 4 5 6 7 8 9 10

None Moderate Complete

##

11. Open a door with a door knob in standing and walking through the doorway (using either

hand)

**____**Yes

## ____NO

____No opportunity

____Unable to perform

____Possibly able to perform, but does not try/avoids task

PERSONAL ASSISTANCE SCALE (C1)

0 Cannot do task or requires the help of 2 or more people

2 Can do task with help of 1 person who does MOST of the work

4 Can do task with help of 1 person who does HALF of the work

6 Can do task with help of 1 person who does LITTLE of the work

8 Can do task but requires SUPERVISION of 1 person

10 Can do task alone

ORTHOTIC SCALE (A1)

0 Cannot do task

1 HKAFO used

2 2 KAFOs used

3 1 KAFO and 1 AFO used

4 1 KAFO used

5 2 AFOs used

6 1 AFO used

7 2 FOs or 1 FO and shoe modifications used

8 1 FO used

9 Shoe modifications used

10 No device used

ENVIRONMENTAL SUPPORT SCALE (B3)

0 Cannot do task

2 Leaned heavily on door frame, assistive device or door

4 Leaned moderately on door frame, assistive device or door

6 Leaned lightly on door frame, assistive device or door

8 Touched lightly for balance only

10 Did not touch door frame, assistive device or door

---------------------------------------------------------------------------------------------

**Functional Performance**

0 1 2 3 4 5 6 7 8 9 10

Cannot do Half Normal

Normal

---------------------------------------------------------------------------------------------

**Confidence**

0 1 2 3 4 5 6 7 8 9 10

None Moderate Complete

12. Wash hands/grooming at the sink in standing

**____**Yes

## ____NO

____No opportunity

____Unable to perform

____Possibly able to perform, but does not try/avoids task

PERSONAL ASSISTANCE SCALE (C1)

0 Cannot do task or requires the help of 2 or more people

2 Can do task with help of 1 person who does MOST of the work

4 Can do task with help of 1 person who does HALF of the work

6 Can do task with help of 1 person who does LITTLE of the work

8 Can do task but requires SUPERVISION of 1 person

10 Can do task alone

ORTHOTIC SCALE (A1)

0 Cannot do task

1 HKAFO used

2 2 KAFOs used

3 1 KAFO and 1 AFO used

4 1 KAFO used

5 2 AFOs used

6 1 AFO used

7 2 FOs or 1 FO and shoe modifications used

8 1 FO used

9 Shoe modifications used

10 No device used

ENVIRONMENTAL SUPPORT SCALE (B3)

0 Cannot do task

2 Leaned heavily on counter / sink or assistive device

4 Leaned moderately on counter / sink or assistive device

6 Leaned lightly on counter / sink or assistive device

8 Touched lightly for balance only

10 Did not touch or lean on counter / sink or assistive device

---------------------------------------------------------------------------------------------

**Functional Performance**

0 1 2 3 4 5 6 7 8 9 10

Cannot do Half Normal

Normal

---------------------------------------------------------------------------------------------

**Confidence**

0 1 2 3 4 5 6 7 8 9 10

None Moderate Complete

1. Reaching into cabinets/closets (above shoulder level, done in standing)

**____**Yes

## ____NO

____No opportunity

____Unable to perform

____Possibly able to perform, but does not try/avoids task

PERSONAL ASSISTANCE SCALE (C1)

0 Cannot do task or requires the help of 2 or more people

2 Can do task with help of 1 person who does MOST of the work

4 Can do task with help of 1 person who does HALF of the work

6 Can do task with help of 1 person who does LITTLE of the work

8 Can do task but requires SUPERVISION of 1 person

10 Can do task alone

ORTHOTIC SCALE (A1)

0 Cannot do task

1 HKAFO used

2 2 KAFOs used

3 1 KAFO and 1 AFO used

4 1 KAFO used

5 2 AFOs used

6 1 AFO used

7 2 FOs or 1 FO and shoe modifications used

8 1 FO used

9 Shoe modifications used

10 No device used

ENVIRONMENTAL SUPPORT SCALE (B3)

**Subtract 2 if reacher was used

0 Cannot do task

2 Leaned heavily on counter, door frame or assistive device

4 Leaned moderately on counter, door frame or assistive device

6 Leaned lightly on counter, door frame or assistive device

8 Lightly touched for balance only

10 Did not touch counter, door frame or assistive device

---------------------------------------------------------------------------------------------

**Functional Performance**

0 1 2 3 4 5 6 7 8 9 10

Cannot do Half Normal

Normal

---------------------------------------------------------------------------------------------

**Confidence**

0 1 2 3 4 5 6 7 8 9 10

None Moderate Complete

14. Retrieving object from floor (from standing position)

**____**Yes

## ____NO

____No opportunity

____Unable to perform

____Possibly able to perform, but does not try/avoids task

PERSONAL ASSISTANCE SCALE (C1)

0 Cannot do task or requires the help of 2 or more people

2 Can do task with help of 1 person who does MOST of the work

4 Can do task with help of 1 person who does HALF of the work

6 Can do task with help of 1 person who does LITTLE of the work

8 Can do task but requires SUPERVISION of 1 person

10 Can do task alone

ORTHOTIC SCALE (A1)

0 Cannot do task

1 HKAFO used

2 2 KAFOs used

3 1 KAFO and 1 AFO used

4 1 KAFO used

5 2 AFOs used

6 1 AFO used

7 2 FOs or 1 FO and shoe modifications used

8 1 FO used

9 Shoe modifications used

10 No device used

ENVIRONMENTAL SUPPORT SCALE (B3)

**Subtract 2 if reacher was used

0 Cannot do task

2 Leaned heavily on assistive device, furniture, etc.

4 Leaned moderately on assistive device, furniture, etc.

6 Leaned lightly on assistive device, furniture, etc.

8 Lightly touched for balance only

10 Did not touch assistive device, furniture, etc.

---------------------------------------------------------------------------------------------

**Functional Performance**

0 1 2 3 4 5 6 7 8 9 10

Cannot do Half Normal

Normal

---------------------------------------------------------------------------------------------

**Confidence**

0 1 2 3 4 5 6 7 8 9 10

None Moderate Complete
