# Supplementary material for: Effects of lower extremity constraint-induced movement therapy on gait and balance of chronic hemiparetic patients after stroke: description of a study protocol for a randomized controlled clinical trial
Source: Trials. 2021 Jul 19;22:463. doi: 10.1186/s13063-021-05424-0 (PMC8287769; doi:10.1186/s13063-021-05424-0)
Supplement: Supplementary file 8 — Additional file 8. [file 13063_2021_5424_MOESM8_ESM.doc]

MANUAL

LOWER EXTREMITY MOTOR ACTIVITY LOG

(LE/MAL)

Constraint-Induced Movement Therapy Research Group

Birmingham VA Medical Center

And University of Alabama at Birmingham

1. **General**

This instrument is a structured interview designed to examine how effectively subjects use their affected leg(s) outside of the laboratory setting. Subjects are asked standardized questions about how much assistance they needed to perform 14 tasks, how well the involved lower extremity(s) performed during the task, and how confident they felt the task could be performed without falling. The subjects are asked to rate each of these factors for 14 functional activities. The rating scales appear in the Scoring Guide, a copy of which should be placed in front of the subject during test administration.

2.  **Rating Scales**

Three scales are used to evaluate performance for each of the 14 tasks. These are the Assistance Scale, the Functional Ability Scale and the Confidence Scale. Each scale is an 11-point Likert scale (0 through 10).

The Assistance Scale consists of 3 sub-scales: A, B, and C. Subscales A and B can take either of two forms, depending on the task. The form of Subscale A (Passive Device-Assistance) relevant to a given task can be either the Orthotic Subscale (A1) or the Equipment Modification Subscale (A2). The scale for Subscale B (Self-Initiated Device-Assistance) relevant to a given task can be either the Assistive Device Subscale (B1) or the Environmental Support Subscale (B2). Subscale C (Person Assistance) is the same for each item. To determine the rating for the Assistance Scale, the tester should obtain scores from the task-relevant A subscale and the task-relevant B subscale and then calculate the average of these 2 scores. Next the test should obtain a score for Subscale C and average this with the mean of (the task-relevant) Subscales A and B. This will yield a score for the Assistance Scale.

Finally, the tester should ask the subject to rate on the other scales (Functional Performance and Confidence) before going on to the next task. Both of these scales are anchored at each end and in the middle by definitions. Refer to the copy of the complete test at the end of these instructions to see the rating scales.

3. **Scoring Guide**

The Scoring Guide is a separate document that contains the scales that the tester should use for scoring each task. It should be used while testing a person. However, the scores should be recorded on the Score Sheet (see below) rather than on the Scoring Guide, since its many pages can then be reused for multiple subjects. As noted, there are two alternate forms of both Subscale A and B. The form most appropriate for each task has been preselected and appears on the scoring guide as a convenience for the tester. For some patients at some points in their treatment, assistance in carrying out an activity may be obtained from sources on more than one of the two forms of Subscale A or Subscale B. For example, a person wearing an orthotic (Subscale B1) may also use an elevated toilet seat (Subscale B2). However, use of an elevated toilet seat is viewed as being of greater assistance for this activity than the orthotic; consequently, it is this form of the subscale (i.e., B2) that should scored for this activity.

Subscales A and B record the extent to which a patient requires assistance from sources other than person(s). Subscale A is designated the Passive Device-Assistance Subscale to indicate that the forms of assistance specified (i.e., orthotics and equipment modifications) provide assistance to persons without any active involvement or their part once they are put in place; i.e., provide passive assistance. Subscale B is designated the Self-Initiated Device-Assistance Scale because the use of the sources of assistance on the scale is self-initiated and optional. The person has to make an effort to use them on every occasion on which they are employed.

The Orthotic Subscale (A1) is used for those activities involving ambulation or that are carried out in standing (i.e., walking, stairs, stepping over objects, turning around, opening door and walking through doorway, washing hands/grooming in standing, retrieving object from floor). The alternate form of the A Subscale, Equipment Modification (A2), is employed for those activities in which the device/equipment used is more important for task accomplishment than an orthotic that the patient may be wearing (i.e., sit-to-stand, lie-to-stand, getting in and out of bath/shower and car).

Subscale C, the Person Assistance Subscale, which characterizes the amount of assistance from another person(s) required to accomplish any activity is scored for each task. This scale indicates the extent to which a patient can carry out an activity without the help of others (i.e., functional independence). Assistance from another person(s) (Subscale C) and assistance from all sources other than another person(s) (Subscales A and B) are viewed as being of equal importance for assessing the level of a person’s ability to carry out an activity. Consequently, in calculating the overall Assistance Score, one first takes a mean of Subscales A and B scores to get an index of the extent to which a person used assistance from all sources other than another person(s) and employs that mean score in the calculation of the overall Assistance from all other sources.

As noted, the Functional Ability and Confidence Scales are 11 point (0-10) Likert scales. They are each anchored by a brief definition at three points; the bottom, top, and mid-point. This laboratory has found that this is adequate to get reliable, internally consistent and accurate indicators of functional status.

4. **Score Sheet**

The score sheet provides a place to enter all of the values needed for arriving at scale, activity and total test score in a brief summary format. No provision is made on the sheet to do the calculation by hand. It is much preferable to enter the values onto a spreadsheet. This not only saves time, but also avoids the arithmetic errors that frequently occur when many calculations must be carried out by hand.

5. **Asking Questions**

**Step One:** The tester should remind the subject that the questions pertain to what they actually do outside the laboratory setting-not what they think they *may be able to do*.

**Step Two:** Time Frame of Questions at Different Test Administrations: The tester should inquire about each activity by asking the following questions:\

1. First Test Administration (screening evaluation)- “Considering your

activities during the past week did you (state the activity)?” If no, write the reason the activity was not performed in the comments section on the score sheet and go to the next activity. If yes, go to step three.

2. Second Test Administration (pre-treatment testing)- “Considering your activities (*one year ago, during the last week*), did you (state the activity)?” If no, write the reason the activity was not performed in the comments section on the score sheet and go to the next activity. In pretreatment testing, information should be obtained about both past week and the one month period one year previously. The tester should go through the questions twice to get the information for the two different time frames. One year in the past, is of course, a long time gap that needs to be bridged in attempting to get accurate information. However, patients with chronic disability normally have a stable motor status over this interval. In addition, one can aid memory by using key, anchoring events such as birthday, holidays, visits to relatives or friends, etc. If the patient’s disability occurred less than two years before, information should be obtained about the month at the one year post-event time point if that was more than six months earlier. If it was less than 6 months earlier (i.e., if patient is less than one and one-half years post-event), only past week data should be obtained.

Note: If the screening evaluation took place less than one week prior to pre-treatment testing, say “Since the time you were evaluated for this project, did you (state the activity)?” If yes go to step 3.

3. All other Administrations- “Since the last time I asked you, did you (state the activity)?” If *no*, check No, check the reason and go to the next activity. If *yes*, go to step three.

**Step Three:** Questions Asked on Different Scales

a. Assistance Scale

1) Subscale A (Passive Device-Assistance)

The tester should say to the subject, “Using the A scale, tell me what orthotics (or equipment modifications) you used to perform (state the activity).” Once the subject selects a rating, repeat the rating and say, “So (or OK), you believe that you used (read the orthotic or equipment modification specified). Is that correct?” Once they agree, the tester should record the response for that question.

2) Subscale B (Self-Initiated Assistive-Device Subscale)

The test should say to the subject, “Using the B scale, tell me what assistive device (or amount of environmental support) you used to perform (state the activity).” Once the subject selects a rating, repeat the rating and say, “So (or OK), you believe that you used (read the assistive device or amount of environmental support specified). Is that correct?” Once, they agree, the tester should record the response for that question.

3) Subscale C (Person Assistance)

The tester should say to the subject, “Using the C scale, tell me how much assistance from another person or persons you needed to perform (state the activity).” The tester should inform the subject of the definitions of different levels of assistance using the Person Assistance Scale. Once the subject selects a rating, repeat the selected rating in the following manner, “So (or OK), you believe that you (read the amount of person assistance specified). Is that correct?” Once they agree, the tester should record the response for that question.

4) The Orthotic (A1), Assistive Device (B1) and Person Assistance (C) Subscales remain the same for each activity for which they are used. However, the Equipment Modification (A2) and Environmental Support (B2) Subscales change from activity to activity (for those activities for which they are used). (See note at end of this document for specific questions and scoring issues for the subscales of the Assistance Scale.)

b. Functional Ability Scale

The tester should say to the subject, “On a scale from ‘0’ to ‘10’, tell me how you would rate your ability to perform (state the activity) now as compared to before the onset of your disability. A ‘0’ means you are unable to do the task. A ‘5’ means that you are able to do the task half as well as before the onset of your disability. A ‘10’ means that you are able to do the task as well as before the onset of your disability. Your rating can be any number from 0 to 10.” Once the subject selects a rating, repeat the selected rating and say, “So (or OK), you believe that you (read their rating to them and tie it to the nearest anchor definition; e.g., 6;”a little more than half is good as before the onset of your disability)-Is that correct?” Once they agree, the tester should record the specified response. Note: For activities that involve predominant use of upper extremities, (11-14): (opening door/walking through doorway, washing hands/grooming in standing, reaching into cabinet/closet, retrieving object from floor), the tester should ask the subject to rate the task on how well the lower extremities functioned during the activity. The function of the upper extremities should not be reflected in the rating score.

c. The tester should say to the subject, “On a scale from ‘0’ to ‘10’, tell me how you would rate your confidence that you can (state the activity) without falling or losing your balance. Remember, you are comparing your confidence to the amount of confidence you had before the onset of your disability. A ‘0’ means you have no confidence that you can (state the task) without falling or losing your balance and therefore, cannot even do the task. A ‘5’ means that you have moderate confidence. A ‘10’ means that you have complete confidence that you can (state the task) without falling or losing your balance.” Once the subject selects a rating, repeat the selected rating and say, “So (or OK), you believe that you (read their answer to them and tie it to the nearest anchor definition; e.g., 8- “somewhat less than complete confidence, but somewhat more than moderate confidence.”) Is that correct?” Once they agree, the tester should record the appropriate response.

**Step Four:** Verify the response

a. During The Pretreatment Test Administration: In this test administration, the tester sets up an agreed-upon rating framework with the subject. If obvious discrepancies exist between what the rater observes and the rating given by the subject, the tester should discuss the rating with the subject to develop a common frame of reference (e.g., You rated that activity a “7”. However, you moved your leg very slowly to do the activity and dropped your foot. So, for this project that would be more like a “5”. Do you agree?”) The final rating must be agreed to by the subject.

Establishing a common frame of reference during the pre-treatment testing, before therapy has begun, is a critically important step. For suggestions on how to accomplish this, see **Comment 1** at the end of this document. The pretreatment administration of the MAL is very important and an hour or more should be devoted to it so that an appropriate frame of reference is established. This should include showing the patient the MAL Demonstration videotape (see Comment 1).

b. During all Other Test Administrations: During testing, the tester should refer back to the immediately previous test administered which should be immediately available to the tester. The form should be out of view of the subject. For example, the previous MAL form might be kept on a clipboard next to the tester, but covered by a piece of cardboard to shield it from the patient’s view. If a rating change occurs, the tester should verify the response by asking the following questions:

1. During the last test, you rated this activity (state either “higher” or “lower”-whichever is accurate) than today by giving it a (repeat the previous score). Why? or Does this represent a real change?

2. So, now that you have thought about it more, how would you rate in now?

3. So you believe that the rating should be (read the rating). Is that accurate? (If yes, record and move on. If no, ask “Why” and go back to question 2.)

**(See comment 2)**

6. **Significant Other Testing**

The LE/MAL should be administered to a significant other on two occasions (on pre-treatment and post-treatment testing days); the patient should not be present during test administration. The same significant other should be tested on both occasions and is preferably someone living with the subject. All rating scales are used both times. At pre-treatment testing the significant other should be asked about past week and past year as described for the patient above.

7. **Calculating the Scores**

The method for calculating the Assistance Scale score for each activity, the activity score, and the total test score may be represented in outline form as follows:

Assistance Scale

a) Subscale A- Passive Device –Assistance Subscale

either A1- Orthotic Subscale

or A2- Equipment Modification Subscale

b) Subscale- Self-Initiated Device-Assistance Subscale

either B1-Assistive Device Subscale

or B2- Environmental Support Subscale

c) Subscale C: Person Assistance Subscale

Assistance Score Calculation

Subscale A + Subscale B + Subscale C

2

__________________________________________________

2

Activity Score Calculation

Assistance + Functional Ability + Confidence

3

Test Score Calculations

1-3) Sum of all individual task scores for a scale

14

Yields a separate score for each scale (i.e., (1) Assistance, (2) Functional Ability, (3) Confidence

4) Composite Lower Extremity MAL score – 1 + 2 +3

3

Missing data: A conservative approach to missing data should be employed. If an activity was inadvertently not scored on a given administration or if the task was not done because there was no opportunity to do it, carry over the score from the previous administration on which it was done.

8. **Specific Questions and Scoring Issues for Assistance Scale**

The following are instructions for specific questions and scoring information:

If different Assistance Devices (Ads) were used at different times for a specific task, then the one used MOST on a given task should be the one that is scored; or if there was a 50/50 ratio, then the lesser score (i.e., poorer performance) should be given. If different amounts of assistance from another person(s) were required for the task during the period of time in questions, the score for the C subscale should be determined by the amount of person assistance that was used MOST. If differing amounts of assistance were used equally, then the lesser score should be recorded for the C subscale.

9. **Walking indoors and walking outdoors**

The distance recorded should be the longest distance the subject walked at one time, without resting, during the period of time in question. The Ads used most during the entire period in question should be recorded, NOT the Ads used when the longest distance was walked. If the subject used a combination of Ads during the period in question, the score should be determined by what AD or combination of them was used the MOST. For, example, if a subject walked indoors using both a straight cane and an AFO 51% of the time, and the AFO only 49% of the time, a score of “6” should be recorded for the A1 (Orthotic) subscale (one AFO used) and a score of “9” should recorded for the B1 (Assistive Device) subscale (one straight cane used). If the ratio of use of different Ads was 50/50, then the lesser score should be recorded. If differing amounts of assistance from a person(s) were required for the task during the period of time in question, the score for the C subscale should be determined by the amount of assistance that was used MOST. If differing amounts of person assistance were used equally, then the lesser score should be recorded for the C subscale.

10.  **Stairs**

The number of stairs recorded would be those climbed up, NOT the combined number negotiated both up and down. The Assistance Score should be determined by what Ads were used the MOST going up and down stairs; or if there was a 50/50 ratio, the lesser score should be recorded. For example, if a subject leaned lightly on one rail and used a AFO going upstairs, and leaned heavily on one rail and used an AFO going down, he/she would score a “6” on the A1 (Orthotic) Subscale and a “4” on the B2 (Environmental Support) Subscale. Further, if the subject went up and down stairs many times while leaning lightly on one rail and on one occasion leaned heavily on one rail, a score of “6” should be given for the B1 (Environmental Support) Subscale. If differing amounts of assistance from a person(s) were required for the task during the period of time in question, the score for the C subscale should be determined by the amount of person assistance that was used MOST. If differing amounts of person assistance were used equally, then the lesser score should be recorded for the C Subscale.

**Comments**

Comment 1: Establishing a context or a common frame of rating reference for the Functional Ability Scale:

a. During the pre-treatment test administration, the subject should be asked to demonstrate a number of activities they have rated on the Functional Ability (FA) Scale. This will allow the tester to get an idea of the subject’s general frame of reference. Demonstration of the performance of an activity should also be requested whenever the tester is unsure of what the subject means by a rating. The demonstration should be carried out after the subject attempts to rate the activity and only when using the FA rating scale. Observation of the demonstration of an activity allows the tester an opportunity to discuss the subject’s FA rating in order to set an agreed-upon rating frame of reference. This process should increase the likelihood that the subject understands the intended motor reference or meaning of the FA rating scale. Therefore, it should increase the comparability of results across subjects. The tester need not have the subject demonstrate every item on the MAL if in their opinion the FA rating is consistent with the performance previously demonstrated on a similar task (e.g., walking indoors and opening a door and walking through doorway). The most useful items to request a demonstration for are those at the beginning of the MAL. Discussion of the subject’s ratings of these items will be helpful in giving them a general idea of what the rating scale means, and they can usually transfer information to aid them in rating later items. However, if there is any question in the tester’s mind, a demonstration of later items should be requested. Establishing a context should be used during pre-treatment testing only, so that experimenter bias and demand characteristics resulting from this procedure cannot artificially increase the apparent extent of a treatment effect.

b. When a clear disparity exists between the subject’s FA rating and what the tester has observed concerning the subject’s motor ability, the tester should explain the meaning of the FA rating scale for the task in question with examples being given for each step, especially those that focus on the FA rating in question (e.g., “You rated that activity a ‘7.’ However, you moved your leg very slowly to do the activity and dragged you foot. So, for this project that would be more like a ‘5.’ Do you agree?”) Subjects will usually be influenced by the tester’s explanation. If they are not and they continue to reiterate the original estimate, the tester should politely continue the discussion until the patient accepts the laboratory/clinic’s frame of reference. Thus, the MAL has aspects of both a self-report instrument and a structured interview. Again, this process should be completed prior to the beginning of treatment to decrease the chance that experimenter bias will influence the FA rating scores.

c. Videotape

This laboratory has developed a videotape that provides examples of different FA rating levels for several MAL activities. This videotape is meant for both the training of clinicians and for establishing a patient common frame of reference for the MAL. Because of the latter part of the intended audience, the description of the movement in the voice-over is kept non-technical. On the video-tape, “% normal” indications are not meant as verictical estimates of the current performance of patients to make judgments of their current ability. It should be shown to the patient at the pre-treatment test administration and discussed thoroughly at that time. During therapy, if the tester believes that the subject’s frame of reference has shifted based on direct observation of behavior, the MAL Demonstration Videotape should be shown again and discussed. A useful rule of thumb would be that if the tester feels that the bias for evaluating performance has shifted more than 4 times, the videotape should be replayed for the patient. The videotape may be obtained by sending a $25 check made out the Constraint-Induced Movement Therapy Research Group to defray the cost of copying and mailing. Send check to:

Edward Taub, PhD

Department of Psychology, 415 CH

University of Alabama at Birmingham

Birmingham, AL 35294

**Comment 2**: Verifying the response after the beginning of treatment

During the standardized questioning, the subjects are not told their previous scores. However, if their report reflects a change in score, whether an increase or a decrease, the change in rating should be probed to determine whether it reflects a true change. The most common type of situation in which probing has been found to increase accuracy sometimes occurs approximately half way through the intervention period. Subjects can become so pleased with their large and rapid improvement in motor function that they tend to magnify and overestimate it. Thus, the majority of errors made by subjects represent an overestimation of the FA rating score. Probing usually results in revisions in the direction of performance decrement (i.e., a lower FA rating score), which therefore leads to a more conservative (and more accurate) estimate of the treatment effect than would otherwise be obtained.

During early experiments in the UAB lab (Taub et. al., 1993), probing was not carried out for the first two patients. The graphs of the daily MAL records for these two patients presented a jagged appearance, with days of decrement following days of large improvement. This variability appeared to tester not to be reflective of the reality of the situation. The improvements were frequently greater than appeared warranted and decrements seemed to underestimate performance. When the probing procedure was implemented, the curve connecting the MAL data points became smooth. However, it is important to note that the final performance was not greater than that recorded for the first two patients (where probing was not used.) The project staff had the clear opinion that the smoothed curves more accurately described subjects’ performance and that this was achieved by eliminating “noise” from the rating process (e.g., lack of attention by subjects during the testing, spontaneous change in the subjects’ frame of reference, etc.).

**Comment 3:** Standard of comparison for Functional Ability Scale

The standard of comparison for making FA rating is the subject’s performance prior to the onset of their disability. Considerable research has shown that in stroke the contralateral arm is not normal, and is therefore not an appropriate final standard of comparison. Appealing to a subject’s general sense of what is normal coordination is probably insufficiently concrete to be useful. On balance, a subject’s memory of his own pre-disability use of the now affected limb(s) is probably the best standard of comparison that can be employed.

# LE MAL

1. Walking indoors

**____**Yes

Distance**:**

____Short distances only/ within room

____Moderate distances/ between adjacent rooms

____Long distances/ length of house, mall, etc.

**____**NO

____No opportunity

____Unable to perform

____Possibly able to perform, but does not try/avoids task

PERSONAL ASSISTANCE SCALE (C1)

0 Cannot do task or requires the help of 2 or more people

2 Can do task with help of 1 person who does MOST of the work

4 Can do task with help of 1 person who does HALF of the work

6 Can do task with help of 1 person who does LITTLE of the work

8 Can do task but requires SUPERVISION of 1 person

10 Can do task alone

ORTHOTIC SCALE (A1)

0 Cannot do task

1 HKAFO used

2 2 KAFOs used

3 1 KAFO and 1 AFO used

4 1 KAFO used

5 2 AFOs used

6 1 AFO used

7 2 FOs or 1 FO and shoe modifications used

8 1 FO used

9 Shoe modifications used

10 No device used

ASSISTIVE DEVICE SCALE (B1)

0 Cannot do task

1 Platform walker (rolling or standard) used

2 Rolling walker used

3 Standard walker used

4 2 forearm crutches used

5 2 quad canes used

6 2 straight canes used

7 1 hemi-walker used

8 1 forearm crutch or 1 quad cane used

9 1 straight cane used

10 No device used

2. Walking outdoors

**____**Yes

Distance**:**

____Short distances/ less than 20 feet

____Moderate distances/ 20 feet to 1 block

____Long distances/ 1 block or more

**____**NO

____No opportunity

____Unable to perform

____Possibly able to perform, but does not try/avoids task

PERSONAL ASSISTANCE SCALE (C1)

0 Cannot do task or requires the help of 2 or more people

2 Can do task with help of 1 person who does MOST of the work

4 Can do task with help of 1 person who does HALF of the work

6 Can do task with help of 1 person who does LITTLE of the work

8 Can do task but requires SUPERVISION of 1 person

10 Can do task alone

ORTHOTIC SCALE (A1)

0 Cannot do task

1 HKAFO used

2 2 KAFOs used

3 1 KAFO and 1 AFO used

4 1 KAFO used

5 2 AFOs used

6 1 AFO used

7 2 FOs or 1 FO and shoe modifications used

8 1 FO used

9 Shoe modifications used

10 No device used

ASSISTIVE DEVICE SCALE (B1)

0 Cannot do task

1 Platform walker (rolling or standard) used

2 Rolling walker used

3 Standard walker used

4 2 forearm crutches used

5 2 quad canes used

6 2 straight canes used

7 1 hemi-walker used

8 1 forearm crutch or 1 quad cane used

9 1 straight cane used

10 No device used

3. Climbing stairs (up and down) ___Number of stairs (one way)

**____**Yes

**____**NO

____No opportunity

____Unable to perform

____Possibly able to perform, but does not try/avoids task

PERSONAL ASSISTANCE SCALE (C1)

0 Cannot do task or requires the help of 2 or more people

2 Can do task with help of 1 person who does MOST of the work

4 Can do task with help of 1 person who does HALF of the work

6 Can do task with help of 1 person who does LITTLE of the work

8 Can do task but requires SUPERVISION of 1 person

10 Can do task alone

ORTHOTIC SCALE (A1)

0 Cannot do task

1 HKAFO used

2 2 KAFOs used

3 1 KAFO and 1 AFO used

4 1 KAFO used

5 2 AFOs used

6 1 AFO used

7 2 FOs or 1 FO and shoe modifications used

8 1 FO used

9 Shoe modifications used

10 No device used

ENVIRONMENTAL SUPPORT SCALE (B3)

0 Cannot do task

2 Leans heavily on 2 rails or 1 rail and assistive device

4 Leans heavily on 1 rail

6 Leans lightly on 1 rail or only uses assistive device on one side

8 Lightly touches rail for balance only

10 No rail or assistive device used

--------------------------------------------------------------------------------------------

**Functional Performance**

0 1 2 3 4 5 6 7 8 9 10

Cannot do Half Normal

Normal

----------------------------------------------------------------------------------------------

**Confidence**

0 1 2 3 4 5 6 7 8 9 10

None Moderate Complete

4. Stepping over object

**____**Yes

**____**No

____No opportunity

____Unable to perform

____Possibly able to perform, but does not try/avoids task

PERSONAL ASSISTANCE SCALE (C1)

0 Cannot do task or requires the help of 2 or more people

2 Can do task with help of 1 person who does MOST of the work

4 Can do task with help of 1 person who does HALF of the work

6 Can do task with help of 1 person who does LITTLE of the work

8 Can do task but requires SUPERVISION of 1 person

10 Can do task alone

ORTHOTIC SCALE (A1)

0 Cannot do task

1 HKAFO used

2 2 KAFOs used

3 1 KAFO and 1 AFO used

4 1 KAFO used

5 2 AFOs used

6 1 AFO used

7 2 FOs or 1 FO and shoe modifications used

8 1 FO used

9 Shoe modifications used

10 No device used

ASSISTIVE DEVICE SCALE (B1)

0 Cannot do task

1 Platform walker (rolling or standard) used

2 Rolling walker used

3 Standard walker used

4 2 forearm crutches used

5 2 quad canes used

6 2 straight canes used

7 1 hemi-walker used

8 1 forearm crutch or 1 quad cane used

9 1 straight cane used

10 No device used

--------------------------------------------------------------------------------------------

**Functional Performance**

0 1 2 3 4 5 6 7 8 9 10

Cannot do Half Normal

Normal

---------------------------------------------------------------------------------------------

**Confidence**

0 1 2 3 4 5 6 7 8 9 10

None Moderate Complete

5. Turning around when standing (whole body with movement of feet)

## ____Yes

**____**No

____No opportunity

____Unable to perform

____Possibly able to perform, but does not try/avoids task

PERSONAL ASSISTANCE SCALE (C1)

0 Cannot do task or requires the help of 2 or more people

2 Can do task with help of 1 person who does MOST of the work

4 Can do task with help of 1 person who does HALF of the work

6 Can do task with help of 1 person who does LITTLE of the work

8 Can do task but requires SUPERVISION of 1 person

10 Can do task alone

ORTHOTIC SCALE (A1)

0 Cannot do task

1 HKAFO used

2 2 KAFOs used

3 1 KAFO and 1 AFO used

4 1 KAFO used

5 2 AFOs used

6 1 AFO used

7 2 FOs or 1 FO and shoe modifications used

8 1 FO used

9 Shoe modifications used

10 No device used

ASSISTIVE DEVICE SCALE (B1)

0 Cannot do task

1 Platform walker (rolling or standard) used

2 Rolling walker used

3 Standard walker used

4 2 forearm crutches used

5 2 quad canes used

6 2 straight canes used

7 1 hemi-walker used

8 1 forearm crutch or 1 quad cane used

9 1 straight cane used

10 No device used

---------------------------------------------------------------------------------------------

**Functional Performance**

0 1 2 3 4 5 6 7 8 9 10

Cannot do Half Normal

Normal

---------------------------------------------------------------------------------------------

**Confidence**

0 1 2 3 4 5 6 7 8 9 10

None Moderate Complete

6. Come to stand from a chair

**____**Yes What type: _______________

## ____NO

____No opportunity

____Unable to perform

____Possibly able to perform, but does not try/avoids task

PERSONAL ASSISTANCE SCALE (C1)

0 Cannot do task or requires the help of 2 or more people

2 Can do task with help of 1 person who does MOST of the work

4 Can do task with help of 1 person who does HALF of the work

6 Can do task with help of 1 person who does LITTLE of the work

8 Can do task but requires SUPERVISION of 1 person

10 Can do task alone

EQUIPMENT MODIFICATION: CHAIR (A2)

0 Cannot do task

2 Used raised chair with armrests

4 Used raised chair without armrests

6 Used standard chair with armrests

8 Used standard chair without armrests

10 Used recliner or soft sofa

UPPER EXTREMITY SCALE (B2)

0 Cannot do task

2 Pushed heavily with both arms

4 Pushed heavily with only one arm or lightly with both arms

6 Pushed lightly with one arm only

8 Lightly touched for balance only

10 Stands without any upper extremity support

---------------------------------------------------------------------------------------------

**Functional Performance**

0 1 2 3 4 5 6 7 8 9 10

Cannot do Half Normal

Normal

---------------------------------------------------------------------------------------------

**Confidence**

0 1 2 3 4 5 6 7 8 9 10

None Moderate Complete

##

7. Come to stand from a toilet

**____**Yes

## ____NO

____No opportunity

____Unable to perform

____Possibly able to perform, but does not try/avoids task

PERSONAL ASSISTANCE SCALE (C1)

0 Cannot do task or requires the help of 2 or more people

2 Can do task with help of 1 person who does MOST of the work

4 Can do task with help of 1 person who does HALF of the work

6 Can do task with help of 1 person who does LITTLE of the work

8 Can do task but requires SUPERVISION of 1 person

10 Can do task alone

EQUIPMENT MODIFICATION SCALE: TOILET (A2)

0 Cannot do task

2 Used elevated toilet with 2 armrests or 2 grab bars

4 Used elevated toilet seat with 1 armrest or 1 grab bar

6 Used elevated toilet seat only or standard height toilet with 2 armrests or 2 grab bars

8 Used stadard height toilet seat with 1 armrest or grab bar

10 Used standard height toilet seat with no arm rests or grab bars

UPPER EXTREMITY SCALE (B2)

0 Cannot do task

2 Pushed heavily with both arms

4 Pushed heavily with only one arm or lightly with both arms

6 Pushed lightly with one arm only

8 Lightly touched for balance only

10 Stands without any upper extremity support

---------------------------------------------------------------------------------------------

**Functional Performance**

0 1 2 3 4 5 6 7 8 9 10

Cannot do Half Normal

Normal

--------------------------------------------------------------------------------------------- **Confidence**

0 1 2 3 4 5 6 7 8 9 10

None Moderate Complete

8. Getting in and out of bed

**____**Yes

____NO

____No opportunity

____Unable to perform

____Possibly able to perform, but does not try/avoids task

PERSONAL ASSISTANCE SCALE (C1)

0 Cannot do task or requires the help of 2 or more people

2 Can do task with help of 1 person who does MOST of the work

4 Can do task with help of 1 person who does HALF of the work

6 Can do task with help of 1 person who does LITTLE of the work

8 Can do task but requires SUPERVISION of 1 person

10 Can do task alone

EQUIPMENT MODIFICATION SCALE: BED (A2)

0 Cannot do task

5 Modified bed used

10 Standard bed used

UPPER EXTREMITY SCALE (B2)

0 Cannot do task

2 Pushed heavily with both arms

4 Pushed heavily with only one arm or lightly with both arms

6 Pushed lightly with one arm only

8 Lightly touched for balance only

10 Stands without any upper extremity support

---------------------------------------------------------------------------------------------

**Functional Performance**

0 1 2 3 4 5 6 7 8 9 10

Cannot do Half Normal

Normal

---------------------------------------------------------------------------------------------

**Confidence**

0 1 2 3 4 5 6 7 8 9 10

None Moderate Complete

##

9. Getting in and out of bath or shower

**____**Yes

## ____NO

____No opportunity

____Unable to perform

____Possibly able to perform, but does not try/avoids task

PERSONAL ASSISTANCE SCALE (C1)

0 Cannot do task or requires the help of 2 or more people

2 Can do task with help of 1 person who does MOST of the work

4 Can do task with help of 1 person who does HALF of the work

6 Can do task with help of 1 person who does LITTLE of the work

8 Can do task but requires SUPERVISION of 1 person

10 Can do task alone

EQUIPMENT MODIFICATION SCALE: TUB (A2)

0 Cannot do task

2 Used tub transfer bench

4 Used tub chair with rail

6 Used tub chair only

8 Used rail only

10 No equipment used

UPPER EXTREMITY SCALE (B2)

0 Cannot do task

2 Pushed heavily with both arms

4 Pushed heavily with only one arm or lightly with both arms

6 Pushed lightly with one arm only

8 Lightly touched for balance only

10 Stands without any upper extremity support

---------------------------------------------------------------------------------------------

**Functional Performance**

0 1 2 3 4 5 6 7 8 9 10

Cannot do Half Normal

Normal

---------------------------------------------------------------------------------------------

**Confidence**

0 1 2 3 4 5 6 7 8 9 10

None Moderate Complete

10. Getting in and out of car

**____**Yes

## ____NO

____No opportunity

____Unable to perform

____Possibly able to perform, but does not try/avoids task

PERSONAL ASSISTANCE SCALE (C1)

0 Cannot do task or requires the help of 2 or more people

2 Can do task with help of 1 person who does MOST of the work

4 Can do task with help of 1 person who does HALF of the work

6 Can do task with help of 1 person who does LITTLE of the work

8 Can do task but requires SUPERVISION of 1 person

10 Can do task alone

EQUIPMENT MODIFICATION SCALE: CAR (A2)

0 Cannot do task

2 Requires sliding board

5 Requires modified seat

10 No modifications required

UPPER EXTREMITY SCALE (B2)

0 Cannot do task

2 Pushed heavily with both arms

4 Pushed heavily with only one arm or lightly with both arms

6 Pushed lightly with one arm only

8 Lightly touched for balance only

10 Stands without any upper extremity support

---------------------------------------------------------------------------------------------

**Functional Performance**

0 1 2 3 4 5 6 7 8 9 10

Cannot do Half Normal

Normal

---------------------------------------------------------------------------------------------

**Confidence**

0 1 2 3 4 5 6 7 8 9 10

None Moderate Complete

##

11. Open a door with a door knob in standing and walking through the doorway (using either

hand)

**____**Yes

## ____NO

____No opportunity

____Unable to perform

____Possibly able to perform, but does not try/avoids task

PERSONAL ASSISTANCE SCALE (C1)

0 Cannot do task or requires the help of 2 or more people

2 Can do task with help of 1 person who does MOST of the work

4 Can do task with help of 1 person who does HALF of the work

6 Can do task with help of 1 person who does LITTLE of the work

8 Can do task but requires SUPERVISION of 1 person

10 Can do task alone

ORTHOTIC SCALE (A1)

0 Cannot do task

1 HKAFO used

2 2 KAFOs used

3 1 KAFO and 1 AFO used

4 1 KAFO used

5 2 AFOs used

6 1 AFO used

7 2 FOs or 1 FO and shoe modifications used

8 1 FO used

9 Shoe modifications used

10 No device used

ENVIRONMENTAL SUPPORT SCALE (B3)

0 Cannot do task

2 Leaned heavily on door frame, assistive device or door

4 Leaned moderately on door frame, assistive device or door

6 Leaned lightly on door frame, assistive device or door

8 Touched lightly for balance only

10 Did not touch door frame, assistive device or door

---------------------------------------------------------------------------------------------

**Functional Performance**

0 1 2 3 4 5 6 7 8 9 10

Cannot do Half Normal

Normal

---------------------------------------------------------------------------------------------

**Confidence**

0 1 2 3 4 5 6 7 8 9 10

None Moderate Complete

12. Wash hands/grooming at the sink in standing

**____**Yes

## ____NO

____No opportunity

____Unable to perform

____Possibly able to perform, but does not try/avoids task

PERSONAL ASSISTANCE SCALE (C1)

0 Cannot do task or requires the help of 2 or more people

2 Can do task with help of 1 person who does MOST of the work

4 Can do task with help of 1 person who does HALF of the work

6 Can do task with help of 1 person who does LITTLE of the work

8 Can do task but requires SUPERVISION of 1 person

10 Can do task alone

ORTHOTIC SCALE (A1)

0 Cannot do task

1 HKAFO used

2 2 KAFOs used

3 1 KAFO and 1 AFO used

4 1 KAFO used

5 2 AFOs used

6 1 AFO used

7 2 FOs or 1 FO and shoe modifications used

8 1 FO used

9 Shoe modifications used

10 No device used

ENVIRONMENTAL SUPPORT SCALE (B3)

0 Cannot do task

2 Leaned heavily on counter / sink or assistive device

4 Leaned moderately on counter / sink or assistive device

6 Leaned lightly on counter / sink or assistive device

8 Touched lightly for balance only

10 Did not touch or lean on counter / sink or assistive device

---------------------------------------------------------------------------------------------

**Functional Performance**

0 1 2 3 4 5 6 7 8 9 10

Cannot do Half Normal

Normal

---------------------------------------------------------------------------------------------

**Confidence**

0 1 2 3 4 5 6 7 8 9 10

None Moderate Complete

1. Reaching into cabinets/closets (above shoulder level, done in standing)

**____**Yes

## ____NO

____No opportunity

____Unable to perform

____Possibly able to perform, but does not try/avoids task

PERSONAL ASSISTANCE SCALE (C1)

0 Cannot do task or requires the help of 2 or more people

2 Can do task with help of 1 person who does MOST of the work

4 Can do task with help of 1 person who does HALF of the work

6 Can do task with help of 1 person who does LITTLE of the work

8 Can do task but requires SUPERVISION of 1 person

10 Can do task alone

ORTHOTIC SCALE (A1)

0 Cannot do task

1 HKAFO used

2 2 KAFOs used

3 1 KAFO and 1 AFO used

4 1 KAFO used

5 2 AFOs used

6 1 AFO used

7 2 FOs or 1 FO and shoe modifications used

8 1 FO used

9 Shoe modifications used

10 No device used

ENVIRONMENTAL SUPPORT SCALE (B3)

**Subtract 2 if reacher was used

0 Cannot do task

2 Leaned heavily on counter, door frame or assistive device

4 Leaned moderately on counter, door frame or assistive device

6 Leaned lightly on counter, door frame or assistive device

8 Lightly touched for balance only

10 Did not touch counter, door frame or assistive device

---------------------------------------------------------------------------------------------

**Functional Performance**

0 1 2 3 4 5 6 7 8 9 10

Cannot do Half Normal

Normal

---------------------------------------------------------------------------------------------

**Confidence**

0 1 2 3 4 5 6 7 8 9 10

None Moderate Complete

14. Retrieving object from floor (from standing position)

**____**Yes

## ____NO

____No opportunity

____Unable to perform

____Possibly able to perform, but does not try/avoids task

PERSONAL ASSISTANCE SCALE (C1)

0 Cannot do task or requires the help of 2 or more people

2 Can do task with help of 1 person who does MOST of the work

4 Can do task with help of 1 person who does HALF of the work

6 Can do task with help of 1 person who does LITTLE of the work

8 Can do task but requires SUPERVISION of 1 person

10 Can do task alone

ORTHOTIC SCALE (A1)

0 Cannot do task

1 HKAFO used

2 2 KAFOs used

3 1 KAFO and 1 AFO used

4 1 KAFO used

5 2 AFOs used

6 1 AFO used

7 2 FOs or 1 FO and shoe modifications used

8 1 FO used

9 Shoe modifications used

10 No device used

ENVIRONMENTAL SUPPORT SCALE (B3)

**Subtract 2 if reacher was used

0 Cannot do task

2 Leaned heavily on assistive device, furniture, etc.

4 Leaned moderately on assistive device, furniture, etc.

6 Leaned lightly on assistive device, furniture, etc.

8 Lightly touched for balance only

10 Did not touch assistive device, furniture, etc.

---------------------------------------------------------------------------------------------

**Functional Performance**

0 1 2 3 4 5 6 7 8 9 10

Cannot do Half Normal

Normal

---------------------------------------------------------------------------------------------

**Confidence**

0 1 2 3 4 5 6 7 8 9 10

None Moderate Complete
