# Supplementary material for: Effects of lower extremity constraint-induced movement therapy on gait and balance of chronic hemiparetic patients after stroke: description of a study protocol for a randomized controlled clinical trial
Source: Trials. 2021 Jul 19;22:463. doi: 10.1186/s13063-021-05424-0 (PMC8287769; doi:10.1186/s13063-021-05424-0)
Supplement: Supplementary file 2 — Additional file 2. [file 13063_2021_5424_MOESM2_ESM.docx]

Reviewer #1: Thanks for this interesting and useful protocol. Some suggestions and recommendations for changes are made below. Numbers refer to the items in the SPIRIT checklist.

We would like to thank the reviewer for the constructive and competent criticism that will certainly improve the methodological description of our paper. We have answered to the suggestions and recommendations followed to each item.

8 What is the framework for the trial eg superiority, non-inferiority?

ANSWER: Once we are working with the same intensity of exercises, we believe that for immediate results the LE-CIT protocol won’t be inferior compared to the conventional therapy.

11c This refers to adherence to the protocol, not necessarily drugs. For example, what measures are in place to promote adherence to the homework that the patients are given.

ANSWER: Patients receive their homework list in a form. For each activity they must check if it was done or not, if they do not perform their task they must write (in the comments space) the reason for not completing the task. They are requested to conduct all the tasks listed; however, they are informed that they must complete at least 70% of the list. Another important aspect is the presence of the caregiver that are supposed to be an encourager for this homework.

For both groups we do not allow more than 2 absences (without medical justifying) during the protocol in order to guarantee the continuing of the protocol (Page 10, line 296-299)

12 This item refers to the outcome measurements to be taken, not the results. These are summarised in table 1 in your manuscript, but could you ensure the primary and secondary outcomes are clearly stated in the measurements section (primary outcome is only mentioned in the sample size section currently), and the analysis metric (eg, change from baseline, final value, time to event) and method of aggregation (eg, median, proportion) for each outcome.

ANSWER: Thanks for the suggestion. We have chosen as primary outcomes the Mini-BESTest and 6mWT (Page 10, line 321-322). WE are going to describe the results of evaluations measure with media and standard deviation for each group, media and standard deviation of differences between groups and confidence interval (Page 14, lines 432-433).

15 Can you give more detail about how you will achieve the required numbers of participants?

ANSWER: AACD is a rehabilitation center, that is a reference center for neurological rehabilitation in Brazil and the main center of rehabilitation in São Paulo, which means that most of the patients who suffered a stroke in São Paulo will be forwarded to AACD. We usually admit for rehabilitation process about 2-4 new patients who suffered a stroke by week. Also, we currently have in therapy about 55 patients who suffered a stroke. All the physiotherapists and the physicians received a check-list with the inclusion criteria when they met patients who attended to these criteria and are available to be part of the protocol. After that they are evaluated for one of our researchers. Only to let you know we’ve had no patients for 3 months because of the pandemic situation. In these regards we are treating the last patient to complete the sample size.

17b Please state why not applicable

ANSWER: Only the evaluators are completely blinded to the interventions. The patients sign the consent form reporting that the group of intervention will not be revealed before the conclusion of the study. One of the exclusion criteria is “do not accept the protocol for which they were randomly allocated”, for sure we cannot guarantee they are blinded once the interventions are different. Actually, we are about to finish the last patient ‘protocol. Therefore, there is no need to unblind it at any point of this time.

18a Do you have a copy of a data collection form or a reference to where one can be found?

ANSWER: We will provide it attached to the documents. We have specific forms for shaping, homework list, LE-MAL and Behavioral contract that we receive at the formation course of LE-CIT protocol. The reference for measure evaluations is in the manuscript.

18b Could you provide this information in the main manuscript, and state what data you intend to collect from patients who don't complete the trial.

ANSWER: Patients receive, at the end of the protocol, the period time they will be asked to return to AACD by 30-15 days before the date we call them by phone contact. In case of giving up from the protocol we will use the completed data of evaluation process until the moment of the renunciation (it has been predicted at informed consent). (Page 10, line 323-326)

19 Please give details about how data will be entered, coded and securely stored

ANSWER: All the collected data are is stored in physical forms; spreadsheet recorded at AACD network where only the researcher can access. The medical record of each patient has a digital and physical format and only the health professionals and the patient can have access.

21a Can you confirm there is no data monitoring committee and if not, why one is not needed?

ANSWER: As we do not have a specific committee for monitoring the data (once we do not have sponsors for this trial) we understand there is no need of a committee. We would like to point out that at AACD we have the ethical committee that can ask for the data and protocol information at any time while the protocol is being carried out at the institution.

23 Can you confirm there will be no trial audit?

ANSWER: As we do not have a sponsor, it was not predicted a trial audit.

26a Please confirm who is responsible for obtaining informed consent

ANSWER: Generally, after accepting being part of our protocol the patient is scheduled for clinical evaluations. In this moment, before starting the evaluation process, the patient receives the consent form, which is read by the main evaluator in the patient’s presence to clarify the possible doubts.

27 Please give more detail on how personal information about potential and enrolled participants will be collected, shared, and maintained in order to protect confidentiality before, during, and after the trial

ANSWER: The personal data have been collected by interviewing the participants and/or their caregiver to collect specific data of stroke and time since stroke. The medical record will be available to the evaluators when necessary. All the collected data are stored in physical forms, spreadsheet recorded at AACD network and only the researchers can access the medical record of each patient (that has a digital and physical format). For statistical analysis the participant’s name will be changed for a number. Therefore, only the main researchers directly involved with the patients will be able to identify them.

30 What, if any, are the provisions for post-trial care or compensation if harms occur?

ANSWER: The possible risks are related to the intensity of the protocol (fatigue and falls), which have been described in the manuscript. If any damage for the patient occurs, the AACD will be responsible for the treatment as predicted for the ethical committee.

Reviewer #2: This is a well designed study that addresses an important disability for persons with stroke.

Thanks for your kind words and suggestions with a special view of clinical aspects that will certainly improve our manuscript and the clinical practice. We have answered to the suggestions and recommendations followed to each item.

Some needed revisions:

1) Inclusion criteria include "gait deficit," but without clarification. How determined?

ANSWER: The first criteria is related to the participant’s perception of gait deficits. So, if there is any complaint about the walking ability related to stroke, the patient will be considered to inclusion in the study. (Page 7, line 186)

2) Similarly, the participant must not have independent gait in the community, but this needs clarification: Can the participant walk independently but using an assistive device?

ANSWER: We defined independent gait in the community as the ability of walking ALONE in their community with or without gait advice, being able to cross streets, achieving at speed of ≥0.8m/s while walking (Page 7, line 198, 199).

3) Among the outcome measures will be the Lower Extremity Motor Activity Log. This is described that the scale evaluates "how effectively subjects use their affected leg outside of the clinic." This is misleading: the LE-MAL assesses quality of bilateral lower extremity use, not only the more-affected leg. This is because the majority of the tasks that the LE-MAL assesses bipedal activities, such as walking, standing, using stairs.

ANSWER: Indeed, the reviewer is right. Most of the activities related to lower limb are bipedal, so LE-MAL was built to evaluate these activities. However, patients are asked to score the quality of movement of his/her affected lower limb while performing the selected task, in the same way they are asked to score his/her confidence on its same leg during this activity (Page 13, line 403-406).

4) As indicated by the abstract, several primary outcome measures will be used, which are performance measures that are conducted in the laboratory: 6-minute walk, timed 10 m walk, timed up and go test, 3D gait analysis, and a balance assessment. To judge the efficacy of a clinical trial, it must have the ability to enroll participants who have deficient performance on these measures. Otherwise, if participants were enrolled at ceiling level of function, it will not be possible to measure an improvement. Yet the inclusion criteria do not specify deficient performance on these measures. Furthermore, if one participant improves primarily on one of these measures but not the others, while a different participant will show a different pattern of improvement (improvement on a different test), how will the study design decide whether it was successful?

ANSWER: We are sorry for the mistake. This information was corrected in the manuscript. The primary outcome chosen were: 6-minute walk test (measure for gait) and Mini-BESTest (measure for balance). The other tests are all secondary (Page 10, line 321-322). We understand that rarely an intervention is going to be superior in all the outcomes chosen.

We’ve chosen Mini-BESTest for balance, first because it is the most complete instrument for balance evaluation, but also because it’s known for not having ceiling effect. Another important point is: we are not choosing them based on the instruments. We’ve chosen them based on clinical criteria (described in inclusion criteria); therefore, they need to have complaints about their ability of walking and not being able to independent walk in community. Based on this information we do not have on our sample patients with mild disability.

5) The LE-MAL is designated as a secondary measure. This study specifically indicates that it aims primarily to assess laboratory-based measures rather than spontaneous lower extremity use as measured by the LE-MAL Yet, stroke participants will most want to understand whether the treatment benefits their community behavior rather than performance under laboratory conditions. The manuscript should explain why this study aims to primarily assess laboratory-based assessments rather than spontaneous lower extremity use on diverse activities in the community.

ANSWER: Unfortunately instruments that are capable of measuring real-world/spontaneous use of lower limb are quite scarce. LE-MAL was not validated for Portuguese-Brazil language (we are working on it). Thus, we do not have financial support to get accelerometers to really evaluate its use (Page 13, line 414-417). In literature these instruments are often chosen to evaluate gait and balance. For our patients, the evaluators give them a feedback by the end of the protocol (for both groups) saying how they have evolved in the period and how it reflected on their participation (based on LE-MAL results). We believe that more important than our measures for giving a feedback to them is their perception of improvement and confidence to realize their daily lives activities.

As we have preliminary results showing that LE-MAL is reliable and valid, it is possible to include it as a primary outcome in the next future with Mini-BESTest and 6mWT.
