# Supplementary material for: Effects of lower extremity constraint-induced movement therapy on gait and balance of chronic hemiparetic patients after stroke: description of a study protocol for a randomized controlled clinical trial
Source: Trials. 2021 Jul 19;22:463. doi: 10.1186/s13063-021-05424-0 (PMC8287769; doi:10.1186/s13063-021-05424-0)
Supplement: Supplementary file 9 — Additional file 9. [file 13063_2021_5424_MOESM9_ESM.docx]

Reviewer #2: This paper summarizes the method of an ongoing clinical trial of lower extremity Constraint-Induced Movement therapy for persons with stroke and gait impairment. The revision has taken into consideration the prior reviews. However, some unresolved concerns:

**1) The Lower Extremity Motor Activity Log will be used, as a secondary outcome measure. This will not be used as a primary outcome measure because at present there is no Portuguese version of this assessment. Despite the availability of the published study on the validation of the LE-MAL by dos Anjos et al (Archives of Physical Medicine and Rehabilitation 2021;102:626-632), the authors do not cite this study, and they state incorrectly that "patients are asked to score the quality of movement of his/her affected lower limb while performing the selected task, in the same way they are asked to score his/her confidence on its same leg during this activity (Page 13, line 403-406)."**

**If they would look carefully at the study by dos Anjos et al 2021, they will see that the LE-MAL does not query the patient on the quality/ confidence of the "more-affected leg," but rather it queries on these qualities in patients who have affected legs. Both sides. There is a misunderstanding that hemiparetic post-stroke patients have a paretic side and the other side is not affected. To the contrast, numerous studies (which concentrated on the upper limbs) have shown that** **the less-affected limb is nonetheless not normal in various kinematic studies.**

**Because the authors plan to depart from the instructions of the use of the LE-MAL by querying the patients of the quality, confidence of the more-affected leg, they need to indicate this departure from the published instructions and why.**

**Answer:** Indeed, we did not cite this paper because our manuscript submission occurred before dos Anjos et al., 2021 study had been published. We have already added this reference on the manuscript (reference 49). Moreover, we are sending (documents attached) the manual that we received at the CIT – course (ministered by David Morris in Brazil - 2016 and David Morris plus Sarah dos Anjos in a second time at UAB - 2019), that we used as reference for applying the instrument. We highlighted in yellow in the manual the description of the LE-MAL: “This instrument is a structured interview designed to examine how effectively subjects use their affected leg(s) outside of the laboratory setting. Subjects are asked standardized questions about how much assistance they needed to perform 14 tasks, how well the involved lower extremity(s) performed during the task, and how confident they felt the task could be performed without falling. The subjects are asked to rate each of these factors for 14 functional activities”.

Thanks for the observation on page 13, line 403-406. We modified the word “affected” to “more-affected”.

Concerning the more-affected leg and the less-affected limb, we understand that when we ask the patient to score confidence and quality of movement comparing to the period before the onset of the disability, they will report about the more affected leg. We hope that this information is clear to satisfy the reviewer question.

**2) The authors plan not to use their primary outcome measures (the 6-minute walk test, the Mini BESTest) as also to be used for the subject inclusion criteria. They state in their reply that it will be sufficient that stroke patients will complain to have of difficulty with walking, and not to use the 6-minute walk test or the Mini BESTest to screen patients for critical impairment on either of these tests.**

**In rigorously designed rehabilitation trials, the primary outcome measures should be used to screen patients for study inclusion, and thus to avoid possibly encountering treatment ceiling effects.**

**Because the authors do not intend to use their primary outcome measures to screen patients, they should indicate that it is possible that patients who self-report having difficulty with walking nonetheless may score highly on these outcome measures and encounter ceiling effects with treatment. Such trial design may limit conclusions of the efficacy of lower extremity Constraint-Induced Movement therapy, and the authors should indicate this study limitation.**

**Answer:** We totally agree with the reviewer that it could potentially be a limitation to this study. However, we would like to clarify that we use the gait speed as inclusion criteria. We excluded only one patient that even having a complaint on gait she was a faster walker (speed for community gait). This patient did not achieve a ceiling effect on Mini BESTest. The paper published by Miyata et al (Phys Ther Res 23: 72-78, 2020) brings us that Mini BESTest has a strong relationship with change in gait speed (r=0.70 and area under the curve= 0.87) and discriminative ability to classify patients in two groups, faster and slower walkers (area under the curve = 0.90). The cut-off for Mini BESTest in this study was 18 for gait speed >0.8 m/s, which means that having the gait speed as criteria of inclusion we will not have ceiling effect on our sample size. In this line, we did not describe in the manuscript as a study limitation.

**3) In the Discussion, the authors query whether it is true that learned misuse can occur in post-stroke hemiparetic patients. It is certainly possible that post-stroke patients may have learned nonuse, as reflected by a reduced amount of walking in the community. However, learned nonuse is also concurrently occurring: In Tani Y, et al, Prevalence of genu recurvatum during walking and associated knee pain in chronic hemiplegic stroke patients: a preliminary survey. J Stroke Cerebrovasc Dis 2016;25:1153-1157, they found that about 20% of stroke patients had this maladaptive movement pattern--a form of learned misuse. Therefore, learned misuse is true. The authors should acknowledge that concurrent learned misuse and nonuse can occur in stroke patients. Lower Extremity CI therapy could be designed to overcome learned misuse as well as learned nonuse.**

**Answer:** We totally agree with the reviewer observation. In the discussion section, our question is about the possibility to argue if we can find ONLY learned misuse, instead of both or predominantly learned nonuse. The first description of the protocol, written by dos Anjos, brings us this idea of presenting misuse instead of nonuse (Phys Ther. 2020 Apr 17;100(4):698-707. doi: 10.1093/ptj/pzz191. PMID: 31899495 – “*The LNU process does not directly apply to the lower extremities because of the bilateral nature of the activities that humans perform with the LEs (e.g., standing, walking) and the fact that the more- affected LE must be used during gait and balance tasks even in the early phase of recovery from injury in order for those activities to be carried out at all, even
though they are carried out incorrectly and ineffectively. Thus, the term “learned misuse” may be a more appropriate term for this situation”*). Nevertheless, in clinical practice we have doubts as just as you notified. We also think that instead of just find exclusively learned misuse we can find both or even only learned nonuse.

**4) Some phrasing should be revised:**

**Under the Abstract, Methods, it states that 42 patients who suffered a stroke "event". The word "event" should be removed (or explain what is the difference between a stroke and a stroke event).**

**The word "followed" is used inappropriately and abundantly in this manuscript. This is not ordinary scientific English in clinical reports (as in for example "The participants ... will receive 15 followed days of daily exercise"), this does not make sense.**

**Answer:** Under the section Assessment of safety and adverse events, the term "TCI" is wrong. Sorry for the mistake. It was corrected to "CIT." Also, the word followed was changed to consecutive and the word event was deleted (in the Abstract section).
